# *Bacillus proteolyticus* OSUB18 triggers induced systemic resistance against bacterial and fungal pathogens in Arabidopsis

**DOI:** 10.3389/fpls.2023.1078100

**Published:** 2023-01-23

**Authors:** Piao Yang, Zhenzhen Zhao, Jiangbo Fan, Yinping Liang, Matthew C. Bernier, Yu Gao, Lijing Zhao, Stephen Obol Opiyo, Ye Xia

**Affiliations:** ^1^ Department of Plant Pathology, College of Food, Agricultural, and Environmental Science, The Ohio State University, Columbus, OH, United States; ^2^ School of Agriculture and Biology, Shanghai Jiao Tong University, Shanghai, China; ^3^ College of Grassland Science, Shanxi Agriculture University, Taigu, China; ^4^ Campus Chemical Instrument Center, Mass Spectrometry and Proteomics Facility, The Ohio State University, Columbus, OH, United States; ^5^ Ohio State University (OSU) South Centers, Piketon, OH, United States; ^6^ Department of Extension, College of Food, Agricultural, and Environmental Sciences, The Ohio State University, Columbus, OH, United States

**Keywords:** *Bacillus proteolyticus*, *Botrytis cinerea*, *Pseudomonas syringae*, *Arabidopsis thaliana*, induced systemic resistance (ISR), callose, reactive oxygen species (ROS), defense priming

## Abstract

*Pseudomonas syringae* and *Botrytis cinerea* cause destructive bacterial speck and grey mold diseases in many plant species, leading to substantial economic losses in agricultural production. Our study discovered that the application of *Bacillus proteolyticus* strain OSUB18 as a root-drench enhanced the resistance of *Arabidopsis* plants against *P. syringae* and *B. cinerea* through activating Induced Systemic Resistance (ISR). The underlying mechanisms by which OSUB18 activates ISR were studied. Our results revealed that the *Arabidopsis* plants with OSUB18 root-drench showed the enhanced callose deposition and ROS production when inoculated with *Pseudomonas syringae* and *Botrytis cinerea* pathogens, respectively. Also, the increased salicylic acid (SA) levels were detected in the OSUB18 root-drenched plants compared with the water root-drenched plants after the *P. syringae* infection. In contrast, the OSUB18 root-drenched plants produced significantly higher levels of jasmonyl isoleucine (JA-Ile) than the water root-drenched control after the *B. cinerea* infection. The qRT-PCR analyses indicated that the ISR-responsive gene *MYC2* and the ROS-responsive gene *RBOHD* were significantly upregulated in OSUB18 root-drenched plants upon both pathogen infections compared with the controls. Also, twenty-four hours after the bacterial or fungal inoculation, the OSUB18 root-drenched plants showed the upregulated expression levels of SA-related genes (*PR1, PR2, PR5, EDS5*, and *SID2*) or JA-related genes (*PDF1.2, LOX3, JAR1* and *COI1*), respectively, which were consistent with the related hormone levels upon these two different pathogen infections. Moreover, OSUB18 can trigger ISR in *jar1* or *sid2* mutants but not in *myc2* or *npr1* mutants, depending on the pathogen’s lifestyles. In addition, OSUB18 prompted the production of acetoin, which was reported as a novel rhizobacterial ISR elicitor. In summary, our studies discover that OSUB18 is a novel ISR inducer that primes plants’ resistance against bacterial and fungal pathogens by enhancing the callose deposition and ROS accumulation, increasing the production of specific phytohormones and other metabolites involved in plant defense, and elevating the expression levels of multiple defense genes.

## Introduction

Food crops are the primary nutrient source of vitamins, minerals, and health-promoting antioxidants worldwide (Giovannoni et al., 2017). Diverse biotic and abiotic stresses could significantly reduce the yield and quality of crops. For instance, under favorable conditions of rainfall and humidity, up to 80% of the fruit crops’ flowers can be lost due to fungal pathogen infections if the fungicides were not used (Ries, 1995). Like the other major food crops, the yield, quality, and security of fruit crops are vastly reduced upon the pathogen infections at the household, national, and global levels (Savary et al., 2019). For example, the fungal pathogen *Botrytis cinerea* leads to gray mold diseases, which could cause significant economic losses to the tomato and strawberry industries (Petrasch et al., 2019). The total annual economic losses of crops caused by *B. cinerea* are over $10 billion globally (Petrasch et al., 2019). Similarly, the bacterial pathogen *Pseudomonas syringae* pathovars caused severe bacterial speck disease in more than 200 plant species, such as the tomato speck disease (pathovar. *tomato*; Shenge et al., 2007) and the bleeding canker of horse-chestnut (pathovar. *aesculi*; Green et al., 2010). Under wet conditions, these disease outbreaks were shown to be devastating (Murillo et al., 2010). For instance, a study reported that bacterial speck disease caused 75% yield losses in field plants infected at their early growth stage (Yunis et al., 1980).

Synthetic chemicals have long been used in the agricultural system to manage plant diseases in order to increase crop yield and improve crop quality (Tronsmo et al., 2020). However, chemical residues pose health risks to humans and wild animals (Fernández-Vizcaíno et al., 2022). More and more restrictions have been placed on the use of synthetic chemicals in crops (Provost and Pedneault, 2016). In addition, overuse of synthetic chemicals could cause pathogen resistance. For instance, copper-based treatments for bacterial pathogens, such as *P. syringae*, are becoming less and less effective due to resistance in bacterial populations (Cazorla et al., 2002). Thus, safer, sustainable, and effective alternatives to synthetic chemicals are urgently needed. Plant growth-promoting rhizobacteria (PGPR) have emerged in agriculture as suitable alternatives to synthetic chemicals for the environmentally safer control of plant diseases (Bhattacharyya and Jha, 2012; Maheshwari et al., 2015). For instance, biological control uses the natural enemies of the pathogens against them, which influences the balance of the plant-microbe interaction. Therefore, the pathogens are eliminated or at least decreased to below economic thresholds (Leppla and LeBeck, 2021). Many *Bacillus* species are popular biological control agents in the agricultural practice (Kumar et al., 2011).

*Bacillus* species are Gram-positive bacteria with rod shapes and are under the phylum of Firmicutes (Turnbull, 1996). Many *Bacillus* species can tolerate different stresses and are potential biological control agents in the agricultural practice (Kumar et al., 2011). For instance, *B. thuringiensis* has been applied as a bio-insecticide because it can produce toxins to kill insects that attach to cotton plants (May, 2011). *B*. *siamensis* can produce antimicrobial metabolites to inhibit plant pathogens, such as *B. cinerea* and *Rhizoctonia solani*, and it can also release volatiles to promote plant growth (Jeong et al., 2012). Some *Bacillus* species, such as *B. amyliliquefaciens*, can not only promote plant growth (Yang et al., 2022) but also secrete certain enzymes as the source of natural antibiotic proteins, including barnase, alpha-amylase, and the BamH1 restriction enzyme (Molohon et al., 2011). *Bacillus simplex* strain WY10 was reported to be able to uptake DNA by transformation (Keen et al., 2017).

Beyond the direct biological control effects, some *Bacillus* spp. have been reported to indirectly benefit plants by triggering an enhanced resistance known as Induced Systemic Resistance (ISR) (Yu et al., 2022). ISR is mediated by beneficial bacteria and fungi living in the rhizosphere. These beneficial microbes impact plant growth and boost plant defenses against phytopathogens and pests (Romera et al., 2019). MYC2 is a transcription factor constitutively expressed in rhizobacteria-induced systemic resistance (Pozo et al., 2008). MYC2 is required for beneficial microbe-triggered ISR and regulates the crosstalk between different phytohormones such as JA, SA, and IAA (Kazan and Manners, 2013). The signaling pathways mediating ISR can differ depending on the plant and microbial species (Ryu et al., 2003; Alizadeh et al., 2013). For example, *Bacillus cereus* C1L activated ISR in tobacco against *B. cinerea* infection *via* the production of volatile dimethyl disulfide (Huang et al., 2012). *Bacillus amyloliquefaciens* FZB42 conferred ISR in *Nicotiana benthamiana* against *B. cinerea* infection *via* inducing stomatal closure and activating SA-, JA/ET-Signaling Pathways (Wu et al., 2018).

*Bacillus proteolyticus* strain OSUB18 was initially isolated from the switchgrass plants through the previously reported approach (Xia et al., 2013). The objectives of this study were to elucidate the mechanisms of OSUB18 on activating the ISR of *Arabidopsis* plants against the pathogens of *P. syringae* and *B. cinerea* by integrated physiological, molecular, and biochemical approaches. Our studies provide new insights into how *Bacillus proteolyticus* could suppress pathogenic bacteria and fungi with different lifestyles by activating the host *Arabidopsis* plant’s systemic resistance through similar but also different defense responses.

## Materials and methods

### Plant materials and growing conditions

All wild-type *A. thaliana* (Col-0) and mutant (*sid2*, *npr1*, *jar1*, and *myc2*) seeds were obtained from the *Arabidopsis* Biological Resource Center (ABRC) in Columbus, Ohio. The homozygous lines were identified based on the information from a published report (Jarret and McCluskey, 2019) and salk website (http://signal.salk.edu/). *A. thaliana* plants were grown in a potting mix (Lambert LM-111) in a walk-in growth chamber (Winnipeg, MB, Canada) at 22 °C with 12-hour light, 12-hour darkness and ~60% relative humidity (Yoo et al., 2022). The light intensity was adjusted as ~120 μmol/m^2^ s (Zhao et al., 2020). Tobacco and tomato seeds were purchased from Park Seed Company at U.S.A. Plants were grown in a professional potting mix (Lambert LM-111) in a greenhouse compartment with air temperatures set at 28 °C, day/night cycles with 16-hour light and 8-hour darkness, and relative humidity at ~45%. Plants were fertilized with Osmocote’s general fertilizer (14-14-4) (Calhoun et al., 2021) to support the needed nutrients.

### Bacterial extracellular exudates collection

OSUB18 was cultured on Tryptic Soy Agar (TSA) plates at 28 °C for 2 days before the bacteria were transferred tosterile 15 mL tubes. 2 mL sterile water was used to wash out all bacterial residues from each TSA plate. TSA plates without OSUB18 were also washed with 2ml sterile water and used as the controls. The 15 mL tubes with OSUB18 cells or controls were homogenized with pipette tips and then transferred to new sterile 2 mL tubes and centrifuged at 12000 rpm for 10 minutes. The above clear cell-free supernatant (CFS) was defined as 1x BEE and stored at -80°C until use. 10x BEE was obtained by concentrating the 1xBEE from 2 mL to 200 µL with a lyophilizer machine. The 5x BEE was diluted two times from the 10x BEE with sterile water.

### Microbial materials and antagonistic assays

OSUB18 was cultured on Tryptic Soy Agar (TSA) plates at 28 °C. Phytopathogenic *P. syringae* pv. tomato DC3000 (*Pst* DC3000) was cultured on King’s B Agar (KBA) plates containing 50 mg/L rifampicin at 28 °C. The grey mold fungus *B. cinerea* was cultured on Potato Dextrose Agar (PDA) plates at 22 °C. Antagonism of OSUB18 against *B. cinerea* was conducted on Potato Soy Agar (PSA, made of PDA and TSA at 1:1 ratio) plates by the dual-culture test. In brief, one agar disc of *B. cinerea* was first placed on one side of the PSA plate. Then, OSUB18 cells (10ul per drop at 10^8 CFU/ml) were spotted on the opposite side away from the fungal plugs in the plates. The plates were then incubated for five days at 22 °C before the fungal growth was measured. The control set was conducted simultaneously by replacing the OSUB18 cells with sterile water. A similar dual-culture assay was carried out to test the direct inhibition effect of OSUB18 against *Pst* DC3000. In brief, *Pst* DC3000 cells (100ul, 10^8 CFU/ml) were evenly distributed on KBA by sterile glass beads (Eyler, 2013). OSUB18 cells (20ul, 10^8 CFU/ml) were spotted to the same plate ~3 cm apart from the plate center. The plates were then incubated for two days at 28 °C before measuring the inhibition zone. The OSUB18/*Pst* DC3000 cells were collected freshly and washed 3 times with sterile water to remove the nutrient residues before use. The above methods were modified from previous reports (Hu et al., 2014; Chen et al., 2018). All experiments were repeated three times with four plates as the replicates for each treatment.

### Phylogenetic assay

The bacterial 16S ribosomal DNA (16S rDNA) sequence of OSUB18 was amplified by polymerase chain reaction (PCR) from its genomic DNA extracted by the Quick-DNA Fungal/Bacterial Micro prep Kit (Zymo Research, Irvine, CA, USA) with the primer pair 799F/1193R (Chen et al., 2020). The purified 16S rDNA sequence was subjected to the Basic Local Alignment Search Tool (BLAST) provided by the National Center for Biotechnology Information (NCBI). We used the Molecular Evolutionary Genetics Analysis (MEGA) software (Tamura et al., 2021) to construct the phylogenetic tree with the Gram positive bacterium *Deinococcus radiophilus* DSM 20551T as an out-cluster control.

### Bacterial phytopathogen infection assay

The leaves of the 4~6-week-old *Arabidopsis* plants were syringe-injected with *Pst* DC3000 at 1 x 10^6^ CFU/mL, dipping inoculated, or spray inoculated with *Pst* DC3000 at 5 x 10^8^ CFU/mL containing 0.05% Silwet L-77 based on the experimental needs. Three days after inoculation, the bacterial growth was quantified by measuring *Pst* DC3000 growth in infected leaves using the serial dilution technique (Katagiri et al., 2002; Yao et al., 2013).

### Fungal phytopathogen infection assay

The leaves of the 4~6-week-old *Arabidopsis* plants were inoculated with *B. cinerea* spores (10ul-drop, 5 x 10^5^ spores/mL in half-strength V8 juice) and covered with a plastic dome to maintain high humidity to facilitate disease development at 22 °C. Three days later, the fungal disease symptom was assessed by measuring the lesion size using the ImageJ software (Weiberg et al., 2013).

### ISR induction by OSUB18

The *A. thaliana* seedlings were transplanted into pots (3.5” x 3.5” x 2”). They were then subjected to a weekly treatment of OSUB18 (10^7^ CFU/mL, 50ml per pot) or water (Ctrl) as the root drench for three consecutive weeks. A week after the last root drench, the treated *A. thaliana*plants were used for bacterial or fungal pathogen inoculation to check ISR responses.

### ROS detection, callose quantification, and ROS burst assay

Superoxide anion (•O_2_^−^) detection by nitroblue tetrazolium (NBT) staining was performed as described by Jambunathan (Jambunathan, 2010). Hydrogen peroxide (H_2_O_2_) detection by diaminobenzidine tetrahydrochloride (DAB) staining was performed based on a previously published method (Nie et al., 2017). Callose deposition was measured following a previous method (Wang et al., 2017). Because the bacterial pathogen was infiltrated into the plant leaves for the infection assay and the fungal pathogen was drop-inoculated on the plant leaves, different patterns of callose deposition were induced. Therefore, they were quantified as callose/mm2 (Jin and Mackey, 2017) in the bacterial infection assay and relative callose (Nie et al., 2017) in the fungal infection assay with Image J software (https://imagej.nih.gov/ij/ ). ROS burst assay was performed following a published protocol (Sang and Macho, 2017).

### Phytohormone extraction and quantification

*A. thaliana* (Col-0) plants were root-drenched with OSUB18 or water (Ctrl) for bacterial or fungal phytopathogen infection assay. At 0 or 24 hours post-infection, the infected leaves were sampled for plant defense-related hormone extraction. SA, SA glucoside (SAG), JA, and JA isoleucine (JA-IIe) were extracted and quantified from ~0.1g fresh weight leaf tissues, as described previously (Zhao et al., 2022b). In brief, phytohormones were extracted from leaf samples with a methanol buffer with the addition of acetic acid and isotope-labeled internal standards (^2^H_4_-SA and ^2^H_2_-JA, CDN Isotopes) on ice. The supernatant was collected after being centrifued at 4 °C and analyzed by UPLC-MS on the Thermo Fisher Ultimate 3000 system (Thermo Fisher Scientific) equipped with a C18 (100 mm × 2.0 mm) column (Waters company, Milford, MA, USA). The solvent gradient used was 100%A (99.9% H_2_O: 0.1% CHOOH) to 100%B (99.9% CH3CN: 0.1% CHOOH) at 200 ul/min. The endogenous and isotope-labeled SA and JA were detected using the following mass transitions: SA 137 > 93, 2H4 SA 141 > 97, SAG 299 > 93, JA 209 > 59, 2H2-JA 211 > 61, JA-IIe 247 > 97.

### Gene expression assay by qRT-PCR

The total RNA of *A. thaliana* (Col-0) leaves with 0 or 24 hours of the bacterial or fungal pathogen infection was extracted with TRIzol Reagent (Invitrogen, Carlsbad, MA, USA). First-strand cDNA was synthesized from 1 µg total RNA with a reverse transcription kit (Applied Biosystem, Waltham, MA, USA). The qRT-PCR was performed on the CFX96 real-time PCR machine (Bio-Rad, Hercules, CA, USA) with *UBIQUITIN10* (*UBQ10*; At4g05320) as the internal reference gene. **Table S1** lists the gene-specific primers. A standard curve was made by determining the threshold cycle (Ct) values for each primer pair’s dilution series of the cDNA product. The gene expression was calculated using the ΔC_T_ method using the *UBQ10* as the reference gene, following the Bio-Rad Real-Time Application Guide. In summary, the relative quantity of each gene is expressed in comparison to *UBQ10* using the formula 2^ (Ct*^UBQ10^*-Ct*^GENE^*), where 2 represents the perfect PCR efficiency. For each qRT-PCR reaction, the Ct was determined by setting the threshold within the logarithmic amplification phase. The ΔC_T_ method is a variation of the Livak (2^-ΔΔCT^) method. The ΔC_T_ method is simpler to perform and gives essentially the same results as the Livak (2^-ΔΔCT^) method (BIO-RAD Real-Time PCR Applications Guide).

### Targeted bacterial metabolites and enzyme activity tests

The O-CAS assay was conducted to detect the bacterial siderophore (Pérez-Miranda et al., 2007). The bacterial exopolysaccharide (EPS) was detected as previously described (Mu’minah et al., 2015). The bacterial acetoin and diacetyl were detected according to the previous report (Khalaf and Raizada, 2018). The bacterial hydrogen cyanide (HCN) was detected according to the previous study (Castric and Castric, 1983). The ammonia production was tested according to the previous report (Vyas et al., 2018).The production of indole acetic acid (IAA) and phosphate solubilization assays were performed according to the previous studies (Maheshwari et al., 2015; Batista et al., 2021).The organic acid was detected on Bromocresol purple Agar (BCPA) with 5′,5″-dibromo-*o*-cresolsulfophthalein as a pH indicator. The catalase activity test was performed according to the previous report (Odds, 1981).All the related experiments had at least 4 replicates and had been repeated 3 times with consistent results.

### Statistical analysis

All statistical analyses were performed using the SPSS software. Data were summarized as mean ± s.e.m unless otherwise stated. Graph bars with different letters or * indicate the p-value < 0.05 by a Student t-test (to compare two groups) or analysis of variance (ANOVA, to compare three or more groups). Data from at least three biological replicates were presented. All experiments were repeated three times to ensure consistency.

## Results

### Isolation and identification of OSUB18 for pathogen inhibition and plant growth promotion

**Figure 1:**
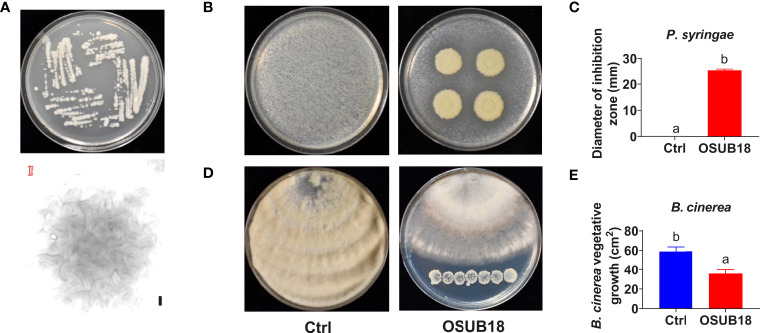
Isolation and identification of OSUB18 for bacterial speck disease and fungal gray mold disease control. **(A)** Colony morphology of OSUB18 on Tryptic Soy Agar (TSA) after 24 hours of incubation at 28°C. The bottom panel is the microscopic image of one representative colony from the upper panel. Scale bar = 100 µm. **(B)** OSUB18 antagonized the bacterial speck disease-causing agent *P. syringae*. **(C)** Quantification of the inhibition zone in **(B)**. **(D)** OSUB18 antagonized the gray mold disease-causing agent *B cinerea*. Note that there was no physical contact between the bacterium and the fungus. **(E)** Quantification of the fungal growth in **(D)**. Data present mean ± SE of three biological replicates. Data with different letters indicate a *p-*value < 0.05 on Student’s t-test.

OSUB18 was initially isolated from switchgrass plants (*Panicum virgatum* L.) in Ohio by the previous method (Xia et al., 2013). We investigated the potential capability of OSUB18 in plant pathogen inhibition and disease control. We noticed that one-day-old OSUB18 cultured on Tryptic Soy Agar (TSA) plates exhibited colonies with undulate margins (**Figure 1A**, upper). The representative crinkle colony was confirmed under a light microscope (**Figure 1A**, bottom). OSUB18 *in vitro* plate assay showed its ability to inhibit the growth of *Pst DC3000* (**Figures 1B, C**), which is the causing agent of bacterial speck disease and the top first plant pathogenic bacterium with a hemibiotrophic lifestyle in molecular plant pathology study (Mansfield et al., 2012). Additionally, we examined the capacity of OSUB18 to control *B. cinerea*, a prevalent fungal pathogen with a necrotrophic lifestyle that causes the destructive grey mold disease on more than 200 plant species (Dean et al., 2012). We found that *in vitro*, OSUB18 significantly inhibited the growth of *B. cinerea* by releasing diffusible functional compounds to the agar media (**Figures 1D, E**). We also found that OSUB18 could increase *Arabidopsis* plants’ shoot biomass and seed yield by the root-drench treatment (**Figure S1**). To characterize OSUB18 at the molecular level, we amplified a PCR fragment from its genome DNA with the internal transcribed sequences (ITS) primers 799F/1193R (Chen et al., 2020) and conducted the Sanger sequencing for the purified PCR fragment. Based on the 16S ribosomal RNA sequences of OSUB18 and the other various sequenced *Bacillus* spp. from the NCBI database, we built a phylogenetic tree for OSUB18. The phylogenetic analysis indicated that OSUB18 belongs most closely to *Bacillus proteolyticus* at the species level (**Figure S2**).

### OSUB18 suppressed bacterial and fungal phytopathogens in multiple plant species

**Figure 2:**
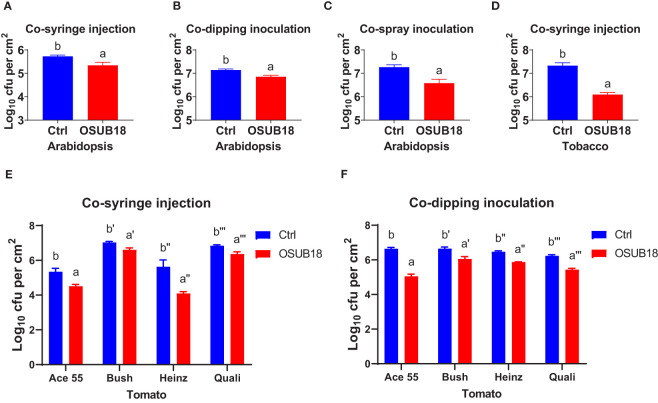
OSUB18 co-inoculation protected plants of different species from the bacterial pathogen *Pst* DC3000 infections. **(A–C)** OSUB18 inhibited *Pst* DC3000 growth in *A thaliana* Col-0 leaves after co-syringe injection **(A)**, co-dipping inoculation **(B)**, or co-spray inoculation **(C)**. **(D)** OSUB18 inhibited *Pst* DC3000 growth in tobacco leaves after co-syringe injection. **(E, F)** OSUB18 inhibited *Pst* DC3000 growth in tomato leaves of diverse cultivars after co-syringe injection **(E)** or co-dipping inoculation **(F)**. An equal volume of water (Ctrl) or OSUB18 was mixed into the *Pst* DC3000 (cell) inoculum for the phytopathogenic infection assay. Data present mean ± SE of three biological replicates. Data with different letters indicate a *p-*value < 0.05 on Student’s t-test.

**Figure 3:**
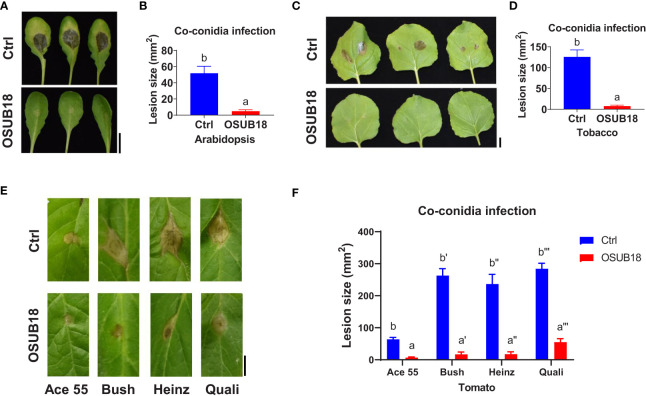
OSUB18 co-inoculation protected plants of different species from the *B cinerea* infections. **(A)** OSUB18 inhibited *B cinerea* growth in *A thaliana* Col-0 leaves after co-inoculation. Scale bar = 10 mm. **(B)** Quantification of the fungal disease symptoms in **(A)**. **(C)** OSUB18 inhibited *B cinerea* growth in tobacco leaves after co-inoculation. Scale bar = 10 mm. **(D)** Quantification of the fungal disease symptoms in **(C)**. **(E)** OSUB18 inhibited *B cinerea* growth in different tomato leaves after co-inoculation. Scale bar = 10 mm. **(F)** Quantification of the fungal disease symptoms in **(E)**. We mixed an equal volume of water (Ctrl) or OSUB18 cells into the *B cinerea* inoculum for the phytopathogenic infection assay. Ace55, Bush, Heinz, and Quali are four popular tomato cultivars in the U.S. market. Data present mean ± SE of three biological replicates. Data with different letters indicate a *p-*value < 0.05 on Student’s t-test.

To further study the efficacy of OSUB18 in antagonizing phytopathogens in plants, we mixed OSUB18 cells or sterile water (control) into the pathogen inoculum and conducted the pathogen infection assay in plants, which is a much more complicated and natural system than the *in vitro* co-culture agar plate assay. As expected, the mixing of OSUB18 in the pathogen inoculum significantly inhibited the growth of *Pst DC3000* in *A. thaliana* Col-0 leaves (**Figures 2A–C**). In addition to the model plant *A. thaliana*, tobacco leaves co-syringe injected with *Pst* DC3000 and OSUB18 showed much weaker disease symptoms (**Figure 2D**) than the water control. In addition, similar results were obtained from 4 different cultivars of tomato plants (**Figures 2E, F** **)**, validating the roles of OSUB18 against the bacterial phytopathogen in different plant species. Likewise, we mingled OSUB18 into the fungal pathogen inoculum of *B. cinerea* for its infection assay. Consistently, the presence of OSUB18 significantly reduced the virulence of *B. cinerea* on Arabidopsis Col-0 plants (**Figures 3A, B**), tobacco plants (**Figures 3C, D**), and tomato plants (**Figures 3E, F**). Our results indicate that OSUB18 has great potential in controlling bacterial and fungal diseases with a broad spectrum in different plant species.

### OSUB18 root drench treatment activated the effective ISR of Arabidopsis against both bacterial and fungal pathogens by enhancing callose deposition and upregulating *MYC2* gene expression

**Figure 4:**
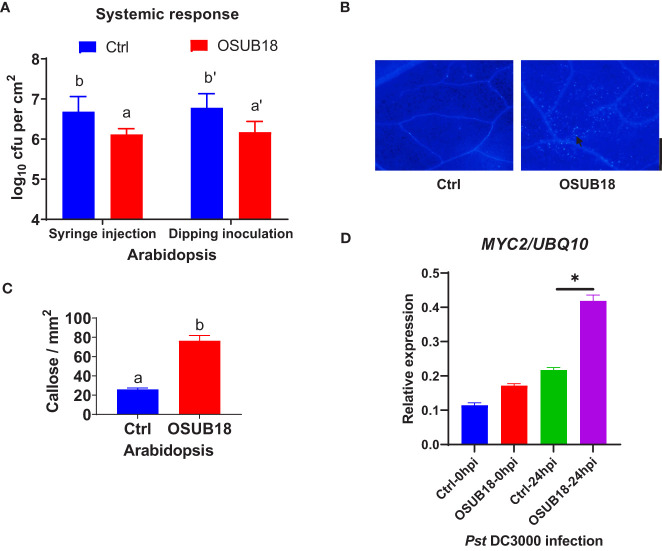
OSUB18 root-drench treatment triggered strong ISR in *A thaliana* against the bacterial pathogen *Pst* DC3000 attack *via* significantly more callose deposition and a significantly higher level of the *MYC2* gene expression. **(A)** OSUB18 root-drench treatment increased the host plant defense of Col-0 plants against *Pst* DC3000 infection. **(B)** Col-0 plants drenched with OSUB18 deposited significantly more callose upon the *Pst* DC3000 infection (24hpi), as illustrated by the aniline blue staining assay. The black arrow indicates a representative callose. Scale bar = 500 µm. **(C)** Quantification of the callose deposition in **(B)**. **(D)** The relative expression level of the ISR-responsive gene *MYC2*. Water or OSUB18-drenched Col-0 plants were infected with *Pst* DC3000 by syringe injection. 0 or 24 hours later, the injected leaves were collected for the qRT-PCR assay. The *UBQ10* gene was used as an internal reference in the qRT-PCR assay. Data present mean ± SE of three biological replicates. Data with different letters or * indicate a *p-*value < 0.05 on Student’s t-test.

**Figure 5:**
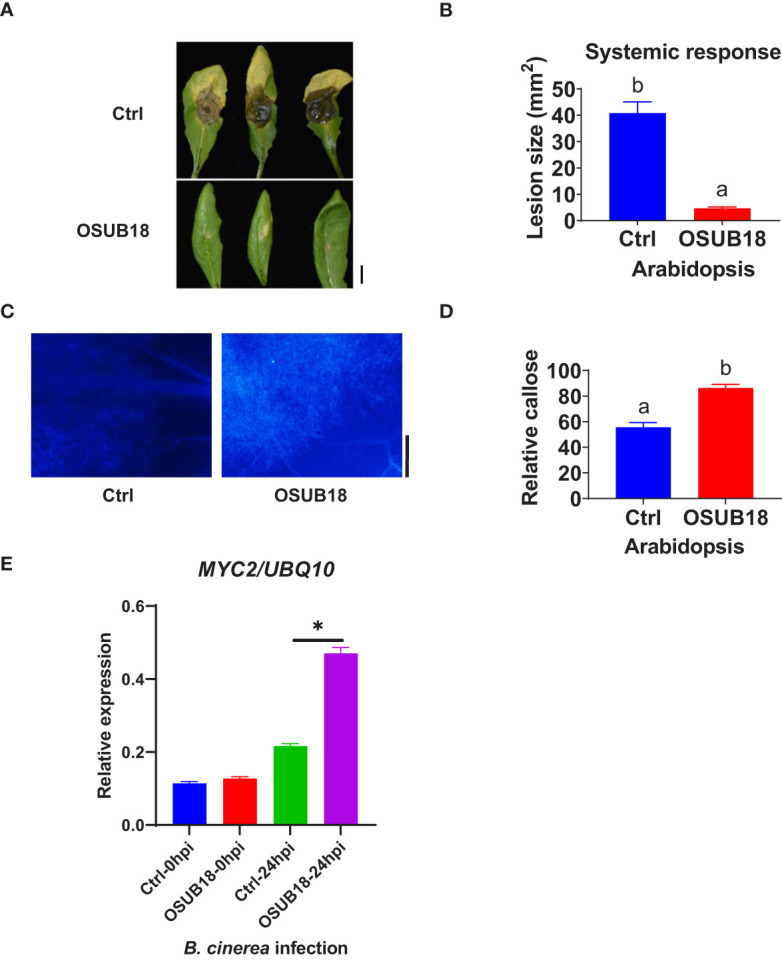
OSUB18 root drench treatment triggered strong ISR in *A thaliana* against the attack of the fungal pathogen *B cinerea via* significantly more callose deposition and higher *MYC2* gene expression. **(A)** OSUB18 root drench treatment increased host plant defense of Col-0 plants against *B cinerea* infection. Scale bar = 5 mm. **(B)** Quantification of the disease symptoms in **(A)**. **(C)** Col-0 plants drenched with OSUB18 deposited significantly more callose upon the *B cinerea* infection (24hpi), as illustrated by the aniline blue-staining assay. Scale bar = 500 µm. **(D)** Quantification of the callose deposition in **(C)**. **(E)** The relative expression level of the ISR-responsive gene *MYC2*. Water or OSUB18-drenched plants were inoculated with *B cinerea* spores. 0 or 24 hours later, the inoculated leaves were collected for the qRT-PCR assay. The *UBQ10* gene was used as an internal reference in the qRT-PCR assay. Data present mean ± SE of three biological replicates. Data with different letters or * indicate a *p-*value < 0.05 on Student’s t-test.

**Figure 6:**
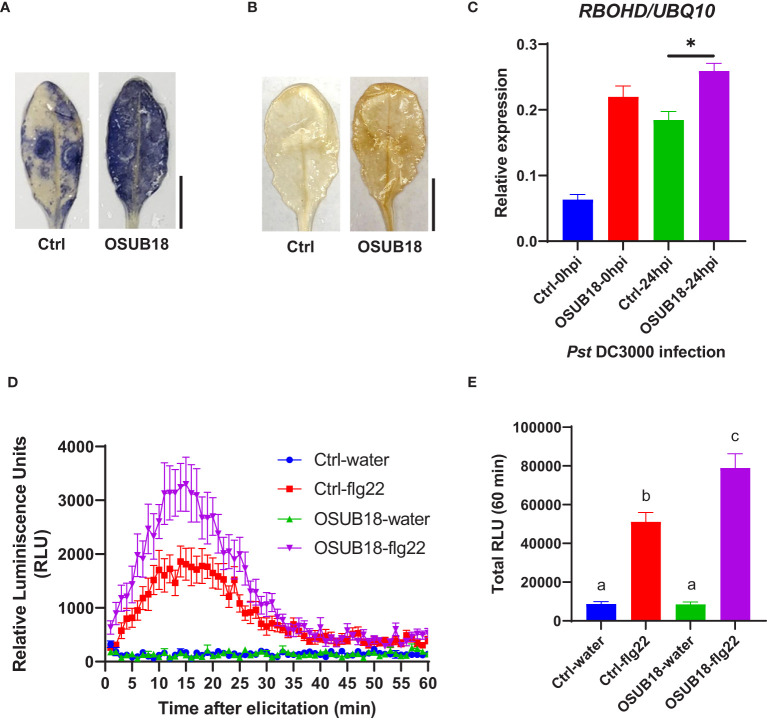
OSUB18 root drench treatment triggered strong ISR in *A thaliana* against the bacterial pathogen *Pst* DC3000 *via* significantly stronger ROS production and higher *RBOHD* gene expression. **(A)** Col-0 plants drenched with OSUB18 produced more intense superoxide anion upon the *Pst* DC3000 infection (24hpi), as illustrated by the nitroblue tetrazolium (NBT) staining assay. Scale bar = 10 mm. **(B)** Col-0 plants drenched with OSUB18 produced more intense hydrogen peroxide upon the *Pst* DC3000 infection (24hpi), as illustrated by the 3,3-diaminobenzidine (DAB) staining assay. Scale bar = 10 mm. **(C)** Relative gene expression of the ROS-responsive gene *RBOHD*. Water or OSUB18-drenched plants were infected with *Pst* DC3000 by syringe injection. 0 or 24 hours later, the injected leaves were collected for the qRT-PCR assay. The *UBQ10* gene was used as an internal reference in the qRT-PCR assay. **(D)** Col-0 plants pre-drenched with OSUB18 showed a more vigorous ROS burst elicited by the bacterial PAMP flg22. **(E)** Quantification of the ROS burst in **(D)**. Data present mean ± SE of three biological replicates. Data with different letters or * indicate a *p-*value < 0.05 on Student’s t-test or ANOVA.

To further investigate the mechanisms of OSUB18 in activating the ISR of host plants against pathogens, we root-drenched *A. thaliana* Col-0 plants with OSUB18 and then challenged their above-ground leaves with the pathogenic bacterium *Pst* DC3000. Both syringe injection and dipping inoculation assays exhibited significantly higher ISR response in *Arabidopsis* against *Pst* DC3000 infection (**Figure 4A**). The leaves were collected 24 hours after the *Pst DC3000* infection to proceed with assays assessing the callose deposition and ISR-responsive gene expression. Compared with control plants (Ctrl, pre-drenched with water), Col-0 plants pre-drenched with OSUB18 accumulated much more callose (**Figures 4B, C**) and significantly higher expression levels of the ISR-responsive gene *MYC2* (**Figure 4D**) upon the *Pst* DC3000 inoculation. Unlike the hemibiotrophic phytopathogens such as *Pst DC3000*, *B. cinerea* has a necrotrophic lifestyle. As a result that OSUB18 antagonized both *Pst DC3000* and *B. cinerea* (**Figures 1**–**3**), we were interested in the ISR efficacy of OSUB18 against phytopathogens with different lifestyles. We thus infected the pre-drenched OSUB18 and water control *A. thaliana* Col-0 plants with *B. cinerea*. We found that Col-0 plants pre-drenched with OSUB18 showed significantly higher resistance against *B. cinerea* infection (**Figures 5A, B**). The Col-0 plants pre-drenched with OSUB18 also exhibited a more robust callose deposition (**Figures 5C, D**) and significantly higher expression of the ISR-responsive gene *MYC2* (**Figure 5E**) against *B. cinerea*. Consistent with previous reports that *MYC2* was a transcription factor constitutively expressed in rhizobacteria-induced systemic resistance (Pozo et al., 2008), our data showed that OSUB18-drenched plants had a remarkably higher expression level of *MYC2* compared to the control plants (**Figures 5E**, **6E**). In agreement with the above results, *Arabidopsis myc2* mutant plants were defective in OSUB18-triggered ISR response against both the bacterial pathogen *Pst* DC3000 (**Figures S5**) and the fungal pathogen *B. cinerea* (**Figure S6**). The result suggested that OSUB18 could successfully activate ISR induction against phytopathogens with different lifestyles.

### OSUB18 root drench treatment activated the ISR of host plants against both bacterial and fungal phytopathogens by enhancing ROS production and upregulating *RBOHD* gene expression

**Figure 7:**
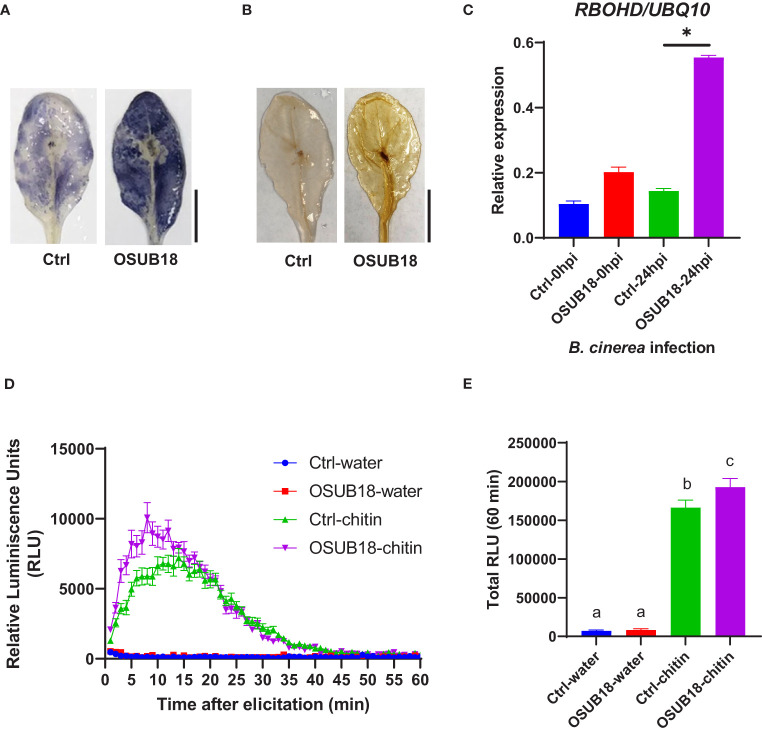
OSUB18 root drench treatment triggered strong ISR in *A thaliana* against the fungal pathogen *B cinerea via* significantly stronger ROS production and higher *RBOHD* gene expression. **(A)** Col-0 plants drenched with OSUB18 produced more intense superoxide anion upon the *B cinerea* infection (24hpi), as illustrated by the nitroblue tetrazolium (NBT) staining assay. Scale bar = 10 mm. **(B)** Col-0 plants drenched with OSUB18 produced more intense hydrogen peroxide upon the *B cinerea* infection (24hpi), as illustrated by the 3,3-diaminobenzidine (DAB) staining assay. Scale bar = 10 mm. **(C)** Relative gene expression of the ROS-responsive gene *RBOHD*. Water or OSUB18-drenched plants were infected with *B cinerea* spores. 0 or 24 hours later, the inoculated leaves were collected for the qRT-PCR assay. The *UBQ10* gene was used as an internal reference in the qRT-PCR assay. **(D)** Col-0 plants pre-drenched with OSUB18 showed a more vigorous ROS burst elicited by the fungal PAMP chitin. **(E)** Quantification of the ROS burst in **(D**). Data present mean ± SE of three biological replicates. Data with different letters or * indicate a *p-*value < 0.05 on Student’s t-test or ANOVA.

Upon the *Pst* DC3000 infection, *Arabidopsis* leaves were collected to proceed with assays assessing the superoxide anion production, hydrogen peroxide production, and ROS-responsive gene expression. Compared with the control plants (Ctrl, pre-drenched with water), Col-0 plants pre-drenched with OSUB18 accumulated much more superoxide anion (**Figure 6A**), hydrogen peroxide (**Figure 6B**), and significantly higher expression of the ROS-responsive gene *RBOHD* (**Figure 6C**), which is essential for the accumulation of ROS in the apoplast (Torres et al., 2002; Zhao et al., 2022a). To verify whether a similar mechanism pattern is present in OSUB18-induced ISR against fungal pathogens, we also infected the pre-drenched OSUB18 *A. thaliana* Col-0 plants with *B. cinerea*. After *B. cinerea* infection, *Arabidopsis* leaves were collected to proceed with assays to assess the superoxide anion production, hydrogen peroxide production, and ROS-responsive gene expression. Indeed, the above-ground leaves of Col-0 plants pre-drenched with OSUB18 also exhibited more robust superoxide anion (**Figure 7A**), hydrogen peroxide (**Figure 7B**), and significantly higher expression level of the ROS-responsive gene *RBOHD* (**Figure 7C**) against the *B. cinerea* infection. To further validate the positive role of ROS production in OSUB18-activated ISR against pathogens in *A. thaliana*, we performed the PAMP-triggered ROS burst assay with leaf discs collected from OSUB18-drenched and water-drenched Col-0 plants. As expected, *A. thaliana* Col-0 plants pre-drenched with OSUB18 showed a significantly stronger ROS burst elicited by the bacterial PAMP flg22 (**Figures 6D, E**) and the fungal PAMP chitin (**Figures 7D, E**) than control plants.

### OSUB18 activated the ISR of *A. thaliana* against the bacterial pathogen *Pst* DC3000 by enhancing its endogenous SA, SAG levels, and expression levels of genes related to the SA-signaling pathway

**Figure 8:**
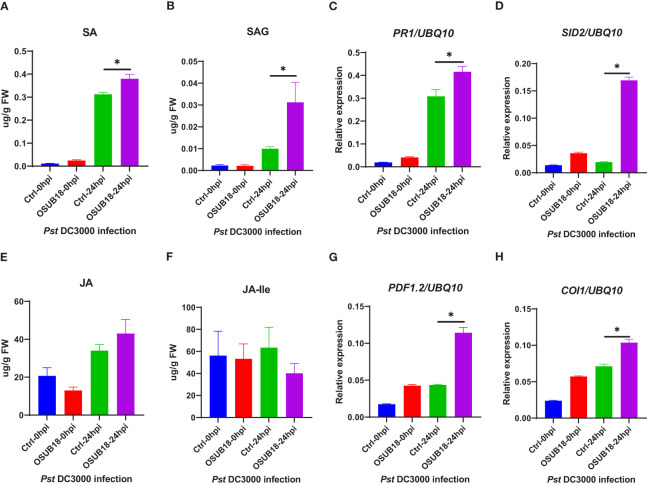
OSUB18 root drench treatment increased the SA and SA-related gene expression levels in *A thaliana* after the bacterial pathogen *Pst* DC3000 infection. **(A)** Free SA level. **(B)** Conjugated SA(SAG) level. **(C)** Relative gene expression of *PR1*. **(D)** Relative gene expression of *SID2*. **(E)** Free JA level. **(F)** Conjugated JA level. **(G)** Relative gene expression of *PDF1.2*. **(H)** Relative gene expression of *COI1*. Water or OSUB18-drenched plants were infected with *Pst* DC3000 by syringe injection. 0 or 24 hours later, the injected leaves were collected for phytohormone quantification and qRT-PCR assay. The *UBQ10* gene was used as an internal reference in the qRT-PCR assay. Data present mean ± SE of three biological replicates. Data with * indicate a *p-*value < 0.05 on Student’s t-test.

To further decipher the underlying mechanism of OSUB18-triggered ISR against the hemibiotrophic phytopathogen *Pst* DC3000, we quantified the levels of SA and SAG in the water- and OSUB18-drenched *A. thaliana* Col-0 plants at 0 hpi and 24 hpi of the *Pst* DC3000 inoculation. We found that the free SA and conjugated SA (SAG) levels were significantly increased in OSUB18-drenched plants 24 hours after the *Pst* DC3000 inoculation compared with the control plants (**Figures 8A, B**). In line with the SA phytohormone production, the representative genes in the SA-signaling pathway (Wang et al., 2019) (*PR1*, *PR2*, *PR5*, *EDS5*, *NPR1* and *SID2*) were significantly upregulated in OSUB18-drenched plants at 24 hours after the *Pst* DC3000 inoculation, compared with the control plants (**Figures 8**, **S3**). There were no significant differences in the JA or JA-lle levels in OSUB18-drenched plants at 24 hours after the *Pst* DC3000 inoculation, compared with the control plants (**Figures 8E, F**), as supported by the normal gene expression levels of *LOX3* and *JAR1* (**Figures S3** **)**. Interestly, the plant JA responsive gene *PDF 1.2* was significantly upregulated after the *Pst* DC3000 inoculation. One of the reasons could be that the *PDF1.2* gene is also regulated by the other upstream genes, such as *NPR1* (Nie et al., 2017). Indeed, we found that the *NPR1* gene was significantly upregulated in the process of OSUB18-induced priming against *Pst DC3000* (**Figure S3D**), which may lead to the increased expression of *PDF 1.2*.In agreement with the above results, *Arabidopsis sid2* and *npr1* mutant plants were defective in OSUB18-triggered ISR response against the bacterial pathogen *Pst* DC3000 but not *jar1* mutant plants (**Figures S5**).

### OSUB18 activated the ISR of *A. thaliana* against the fungal pathogen *B. Cinerea* by enhancing its endogenous JA and JA-lle levels and upregulating genes related to the JA-signaling pathway

**Figure 9:**
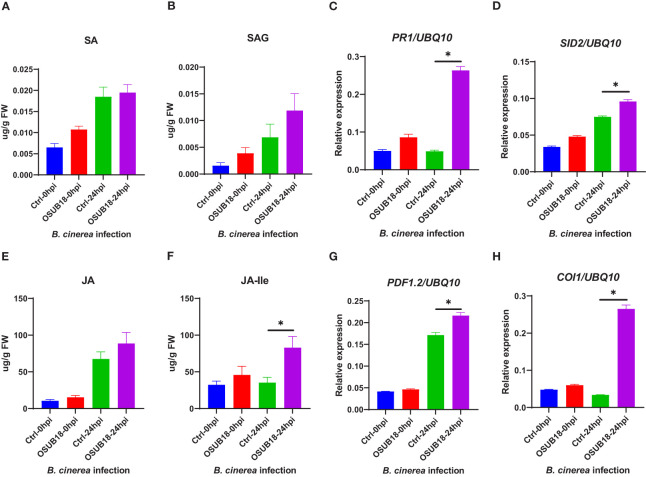
OSUB18 root drench treatment increased the JA and JA-related gene expression levels in *A thaliana* after the fungal pathogen *B cinerea* infection. **(A)** Free SA level. **(B)** Conjugated SA (SAG) level. **(C)** Relative gene expression of *PR1*. **(D)** Relative gene expression of *SID2*. **(E)** Free JA level. **(F)** Conjugated JA level. **(G)** Relative gene expression of *PDF1.2*. **(H)** Relative gene expression of *COI1*. Water or OSUB18-drenched plants were infected with *B cinerea* by spore inoculation. 0 or 24 hours later, the injected leaves were collected for phytohormone quantification and qRT-PCR assay. The *UBQ10* gene was used as an internal reference in the qRT-PCR assay. Data present mean ± SE of three biological replicates. Data with * indicate a *p-*value < 0.05 on Student’s t-test.

To further examine the underlying mechanism of OSUB18-triggered ISR against the necrotrophic phytopathogen, we quantified the levels of JA and JA-lle in the water- and OSUB18-drenched *A. thaliana* Col-0 plants at 0hpi and 24hpi of the *B. cinerea* inoculation. Higher JA levels (**Figures 9E**) and significantly higher levels of JA-lle (the bioactive form of jasmonate) were detected in OSUB18-drenched plants at 24 hours after the *B. cinerea* inoculation, compared with the control plants (**Figures 9F**). Consistent with the phytohormone quantification results, the representative genes in the JA-signaling pathway (*PDF1.2*, *COI1, LOX3*, and *JAR1*) and the main ISR regulator *NPR1* were significantly upregulated in OSUB18-drenched plants at 24 hours after the *B. cinerea* inoculation, compared with the control plants (**Figures 9**, **S4**). In agreement with the above results, *Arabidopsis jar1* and *npr1* mutant plants were defective in OSUB18-triggered ISR response against the fungal pathogen *B. cinerea but* not *sid2* mutant plants (**Figures S6**). Though the representative genes in the SA-pathway (*PR1*, *PR2, PR5*, *SID2*) were also upregulated (**Figures 9C, D**, **S3**), there were no significant differences in the SA or SAG levels in OSUB18-drenched plants 24 hours after the *B. cinerea* inoculation, compared with the control plants (**Figures 9A, B**). Possibly, it is due to the downregulated expression of *EDS5* (**Figure S4**), which is responsible for the transport of the SA precursor isochorismate (IC) from chloroplast to cytosol for the SA biosynthesis in cytosol.

### OSUB18 exhibited diverse plant protection and growth promotion traits by producing different compounds and enzymes

**Figure 10:**
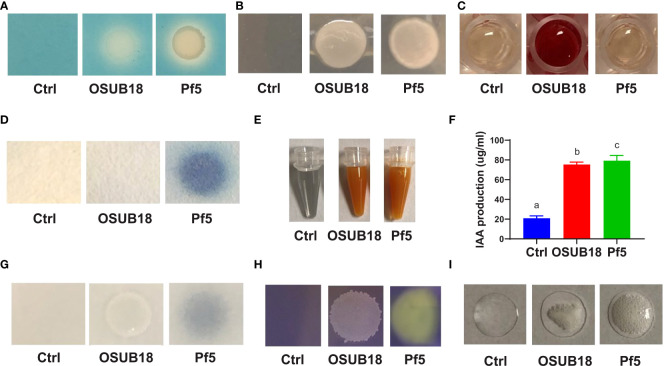
OSUB18 produced beneficial ISR-inducing metabolites suppressing plant pathogens and diseases and promoting plant growth. **(A)** Siderophore production. **(B)** Exopolysaccharide production. **(C)** Acetoin and diacetyl production. **(D)** HCN production. **(E)** Ammonia production. **(F)** IAA production. **(G)** Phosphate solubilization. **(H)** Organic acid production. **(I)** Catalase activity. The plant commensal bacterial *Pseudomonas fluorescens* strain Pf-5 was used as a control. Data present mean ± SE of three biological replicates. Data with different letters indicate a *p-*value < 0.05 on ANOVA.

Some chemical compounds, such as siderophores, polysaccharides, and volatile acetoin/diacetyl, are critical in ISR elicitation (Berendsen et al., 2015; Jiang et al., 2016). For our study, we found that OSUB18 was positive in producing siderophores (**Figure 10A**), exopolysaccharides (**Figure 10B**), and acetoin/diacetyl (**Figure 10C**). These data aligned with OSUB18’s efficacy in activating ISR against both bacterial and fungal phytopathogens (**Figures 4**, **5**). OSUB18 was positive in producing acetoin/diacetyl (**Figure 10C**) but not HCN (**Figure 10D**), suggesting acetoin/diacetyl-related compounds can be the functional VOCs, in contrast to *Pseudomonas fluorescens* strain Pf5, one well-known bacterial strain involved in ISR. The production of ammonia by OSUB18 (**Figure 8E**) may also provide host plants with an additional nitrogen source for growth and defense. The phytohormone auxin could promote plant shoots’ and lateral roots’ growth (Woodward & Bartel, 2005). The enhanced shoot growth of OSUB18-inoculated plants (**Figures S1C, D**) is consistent with the fact that OSUB18 could produce ample amounts of IAA (**Figure 10F**). However, OSUB18 did not solubilize phosphate (**Figure 10G**) nor produce organic acid (**Figure 10H**), suggesting its plant growth-promoting role might be independent of these two factors. The catalase activity of OSUB18 (**Figure 10I**) may also play a positive role in protecting host plants by increasing oxygen availability in potting mix/soil and facilitating the gas exchange of plants with the environment. In agreement with the above beneficial traits, OSUB18 metabolites (cell-free crude extracts) inhibited the growth of *Pst* DC3000 (**Figure S7A**) and prevented the spore germination and hypha development of *B. cinerea* (**Figure S7B**).

## Discussion

OSUB18 was isolated from switchgrass and further characterized due to its capacity to promote plant growth (**Figure S1**). In this study, we investigated the roles and related mechanisms of OSUB18 in activating the ISR of *Arabidopsis* plants against diverse pathogens, including *Pst DC3000* and *B. cinerea.* We discovered that OSUB18 antagonized bacterial and fungal phytopathogens both in-vitro plate assay (**Figure 1**) and in planta (**Figures 2** **,** **3**) in *Arabidopsis*, tobacco, and tomato plants. Additionally, OSUB18 root treatment primed vigorous ISR activities of *Arabidopsis* plants against the bacterial phytopathogen *Pst DC3000* (**Figure 4**) and the fungal phytopathogen *B. cinerea* (**Figure 5**). These data suggest that OSUB18 could be an excellent biological control agent with a broad-spectrum efficacy against bacterial and fungal phytopathogens *via* both direct antagonism and indirect ISR mechanisms in different plant species.

Plant leaves use cuticles and cell walls as the first barrier to avoid pathogen attacks (Malinovsky et al., 2014). For example, plants evolved to deposit polymers, such as callose, phenolic compounds, and antimicrobial toxins at the cuticle and cell walls to defend against pathogen infections (Hückelhoven, 2007; Zhao et al., 2020; Ziv et al., 2018). PAMPs, such as flg22 and chitin, can induce callose deposition, which was thus widely used to evaluate the plant PTI (Luna et al., 2010) by aniline blue staining (Jin and Mackey, 2017). In this study, we found that the callose deposition was significantly higher in OSUB18 root-treated plants than in water-treated plants upon the bacterial (**Figures 4B, C**) or fungal (**Figures 5C, D**) pathogen infections, indicating that cell wall reinforcement contributed to the OSUB18 associated ISR. Additionally, we found that the gene expression of transcription factor *MYC2* was significantly higher in OSUB18-treated plants than that in the water-treated plants, consistent with the previous report showing that *MYC2* could be involved in priming plant defense in the rhizobacterial-activated ISR of *Arabidopsis* (Pozo et al., 2008).

Reactive oxygen species (ROS) play central roles in plant signaling and immunity *via* diverse cellular processes (Qi et al., 2017). Plants evolved to harness toxic and signaling properties of ROS to defend themselves against invading pathogens (O’Brien et al., 2012). For example, plant plasma membrane-localized respiratory burst oxidase homologs (RBOHs) produce apoplastic ROS in response to pathogen infections. RBOHD is the most well studied member of the 10 RBOHs in *Arabidopsis* that plays essential roles in plant immunity by producing hydrogen peroxide (H_2_O_2_) (Kadota et al., 2015). In this study, we found that during the defense priming, OSUB18-treated plants exhibited significantly higher ROS production and gene expression of *RBOHD* than those in water-treated plants for both hemibiotrophic and necrotrophic pathogen infections (**Figures 6**, **7**). These results indicate that ROS contributed to OSUB18-activated ISR.

Phytohormones play vital roles in plant development and defense (Bari and Jones, 2009; Santner et al., 2009). However, whether phytohormone pathways contribute to the OSUB18-activated ISR remains unclear. In this research study, we revealed that under the attacks of the hemibiotrophic bacterial pathogen *Pst DC3000*, SA levels were significantly higher in OSUB18-treated plants than in the water-treated plants at 24 hpi (**Figure 8A**). On the other hand, upon the attacks of the necrotrophic fungal pathogen *B. cinerea*, JA-lle (the bioactive form of JA) levels were significantly higher in OSUB18-treated plants than those in the water-treated plants at 24 hpi (**Figure 9F**). In line with the phytohormone quantification results, we detected the related gene expression by qRT-PCR and found that SA pathway-related genes, such as *PR1, PR2, PR5, EDS5, NPR1*, and *SID2*, were significantly induced in OSUB18-treated plants against the hemibiotrophic bacterial pathogen *Pst DC3000* (**Figures 8**, **S3**).However, JA pathway-related genes, such as *PDF1.2, LOX3, JAR1*, and *COI1* were significantly induced in OSUB18-treated plants against the necrotrophic fungal pathogen *B. cinerea* (**Figures 9**, **S4**), compared with water-treated plants. These data indicate that SA and JA contributed to the OSUB18-activated ISR against various pathogen infections, which depends on the pathogen’s lifestyles.

In addition, the representative genes in the SA pathway (*PR1*, *PR2*, *PR5*, *SID2*) were also upregulated, but no significant differences in the SA or SAG levels were found in OSUB18-drenched plants after the *B. cinerea* infection (**Figure 9**). One of the reasons could be that many genes are involved in the final outcome of the SA or SAG levels. For instance, among those genes involved in SA biosynthesis and metabolism, *EDS5* controls the outport of SA precursor isochorismate (IC) from chloroplast to cytosol for the SA biosynthesis (Ding and Ding, 2020). We found that the experession level of *EDS5* was significantly downregulated compared with the control in the case of *B. cinerea* infection (**Figure S4C**), suggesting a decreased IC pool in the cytosol and, thus leading to a none significant increase in the final SA or SAG levels. The other possible reason is that *PR* genes can be regulated by the other upstream genes. For instance, *NPR1* is a crucial defense gene downstream of SA and JA but upstream of *PRs* and *PDF1.2* in ISR against both biotrophic and necrotrophic phytopathogens (Nie et al., 2017). Indeed, we found that the *NPR1* gene and its downstream *PR* genes (*PR*1, *PR2, PR5*) were significantly upregulated in OSUB18-drenched plants after the *B. cinerea* infection compared with the control plants (**Figure S4**). In agreement with the above results, *Arabidopsis jar1* and *npr1* mutant plants were defective in OSUB18-triggered ISR response against the fungal pathogen *B. cinerea* but not *sid2* mutant plants (**Figures S6**).

We also noticed that the level of JA showed no significant change, but the expression of JA-related genes, such as *PDF 1.2*, were significantly changed in the process of OSUB18-induced priming against *Pst* DC3000 (**Figure 8**). One of the reasons could be that the *PDF1.2* gene is also regulated by the other upstream genes, such as *NPR1*. As mentioned above *NPR1* is a crucial defense gene upstream of *PRs* and *PDF1.2* in ISR against plant pathogens with different lifestyles (Nie et al., 2017). Indeed, we found that the *NPR1* gene was significantly upregulated in the process of OSUB18-induced priming against *Pst* DC3000 (**Figure S3D**). The other possible reason is that many genes are involved in the final outcome of the JA level. Among those genes involved in JA biosynthesis and metabolism, *LOX3* converts 18:3 to the JA precursor OPDA in the chloroplast, and JAR1 converts JA to JA-lle, the bioactive form of JA in the cytoplasm (Ruan et al., 2019; Habash et al., 2020). We found that the expression leves of *LOX3* and *JAR1* did not significantly change in the process of OSUB18-induced priming against *Pst* DC3000 (**Figure S3**), suggesting a normal JA pool. In agreement with the above results, *Arabidopsis sid2* and *npr1* mutant plants were defective in OSUB18-triggered ISR response against the bacterial pathogen *Pst* DC3000 (**Figure S5**) but not *jar1* mutant plants. Indeed, increasingly studies confirmed that the favorable signal transduction pathways during ISR not only depend on the host plants and beneficial microbes but also the pathogen lifestyles (Shoresh et al., 2010; Pieterse et al., 2014; Yu et al., 2022).

Microbial secondary metabolites function differently and diversely in biological activities, such as (Leong, 1986; Makras and De Vuyst, 2006; Kai et al., 2007; Mota et al., 2017; Anand et al., 2020; Abdalla et al., 2021), plant growth (Hayat et al., 2010; Etesami et al., 2015; Rijavec and Lapanje, 2016; Pahari et al., 2017; Alori et al., 2017; Naseem et al., 2018; Sharifi and Ryu, 2018; Macias-Benitez et al., 2020; Lopes et al., 2021), and insecticidal activities (Ullah et al., 2020). Interestingly, many traditional microbial secondary metabolites have recently been reported to play novel roles in (Berendsen et al., 2015; Jiang et al., 2016; Peng et al., 2019). For instance, *Bacillus velezenisis* was recently engineered to produce higher acetoin to prime a strong ISR in *Arabidopsis* (Peng et al., 2019), though acetoin was well-known for its volatile and antagonistic properties (Kai et al., 2007). Our study found that OSUB18 produced several secondary metabolites (siderophore, exopolysaccharide, and acetoin) reported to be ISR inducers (**Table S2**), partially explaining the promising activities of OSUB18 in ISR. OSUB18 was also positive in producing secondary metabolites, such as ammonia, with reported roles in pathogen inhibition (Mota et al., 2017). This might partially explain why OSUUB18 inhibited bacterial and fungal pathogen growth *in vitro* (**Figure 1**) and planta (**Figures 2**, **3**). In addition, OSUB18 produced growth hormone IAA (**Figure 10**) with the reported roles in plant growth promotion (**Table S2**), which may contribute to plant growth activation. Although our work extends the plant protection properties of OSUB18 from switchgrass to *A. thaliana*, tobacco, and tomato (**Figures 2**, **3**), little is known about the modes of action of OSUB18 in promoting plant growth. Recently, VOCs produced by PGPRs have been reported to induce ISR against phytopathogens and promote plant growth (Lee et al., 2012; Pérez-Flores et al., 2017). For example, the volatile 2,3-butanediol and 3-hydroxy-2-butanone (acetoin) were found in multiple beneficial bacteria, including *Bacillus* spp. (Effantin et al., 2011; Yi et al., 2016). Applying these ISR-eliciting VOCs to tobacco plants has been reported to increase host resistance against the fungal pathogen *Colletotrichum orbiculare*  (D’alessandro et al., 2014).

In summary, our study discovered that OSUB18 is a novel strain that can activate plant ISR against diverse pathogens by enhancing the callose deposition, ROS accumulation, SA or JA-lle production, and the other ISR related signaling pathways in *Arabidopsis* plants. Further studies need to be conducted to reveal the underlying mechanisms of OSUB18-activated ISR in other plant species. In addition, investigating the persistence and propagation of OSUB18 in plants grown in potting mix or soils under different environmental conditions can yield critical information for its applications in agricultural practice. The results from these studies could deepen our understanding of the underlying mechanisms of plant ISR activated by the beneficial microbes, facilitate the application of these beneficial microbes in agriculture practice to protect plants against pathogens, increase crop yields, and improve food quality while lowering pesticide input.

## Data availability statement

The original contributions presented in the study are included in the article/**Supplementary Material**. Further inquiries can be directed to the corresponding author.

## Author contributions

PY and YX conceived and designed the experiments. PY performed the experiments. YG helped with the drafting and editing of the manuscript. All authors developed experimental approaches or analyzed the data. PY and YX wrote the manuscripts with help from all the authors. All authors approved the final version of the manuscript.
