# Supplementary material for: Bacillus proteolyticus OSUB18 triggers induced systemic resistance against bacterial and fungal pathogens in Arabidopsis
Source: Front Plant Sci. 2023 Jan 23;14:1078100. doi: 10.3389/fpls.2023.1078100 (PMC9900001; doi:10.3389/fpls.2023.1078100)
Supplement: Supplementary file 1 [file Presentation_1.pdf]

Supplementary Material

Figures S1-S7
Tables S1-S2

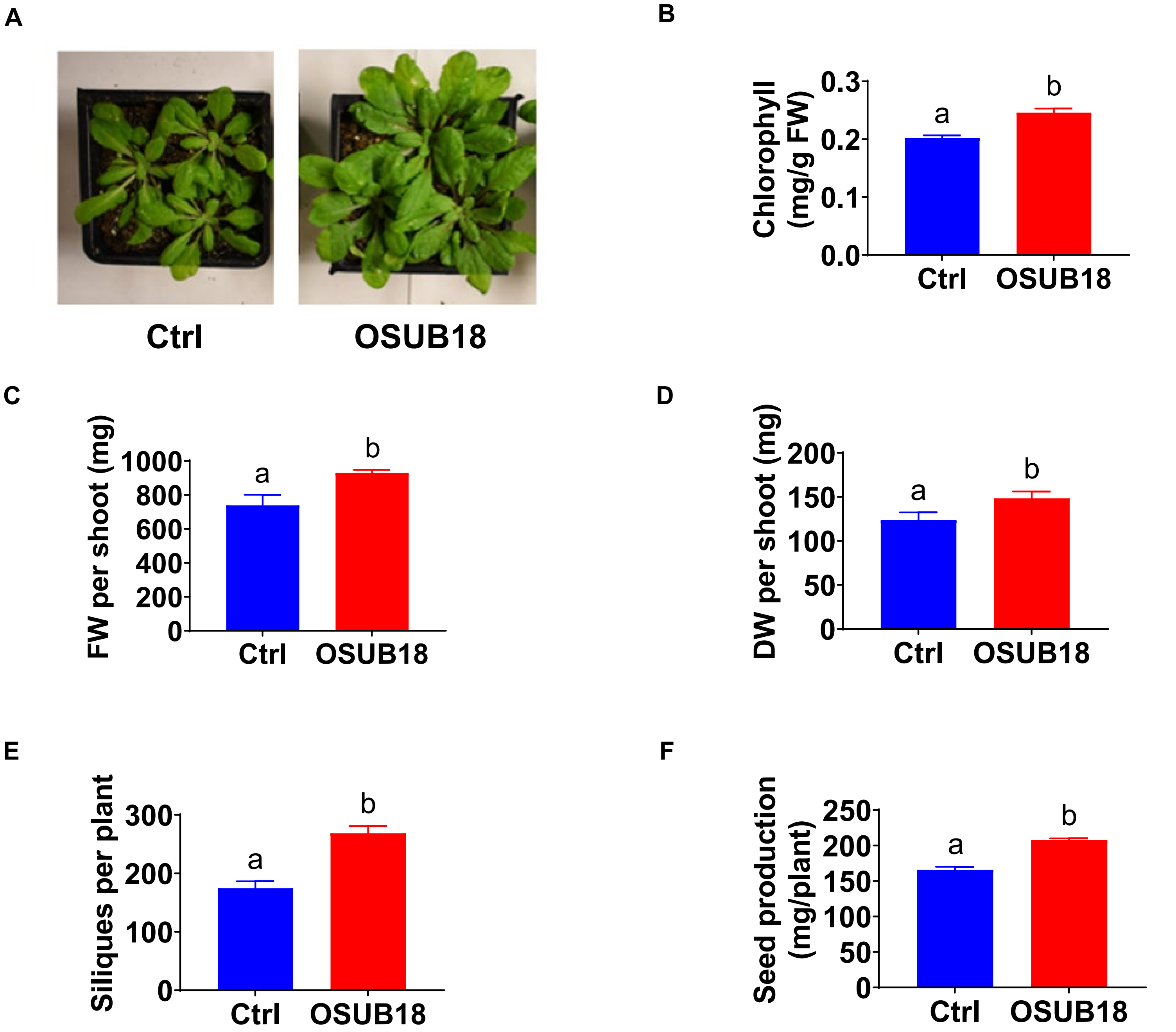

FIGURE S1

FIGURE S1 | OSUB18 promoted the growth and yield of *A. thaliana* plants. (A) OSUB18 root-drench treatment promoted the growth of Col-0 plants. **(B-E)** Quantification of the leaf chlorophyll level **(B)**, shoot fresh weight (FW) **(C)**, shoot dry weight (DW) **(D)**, silique production **(E),** and seed production **(F)** of wide-type Col-0 plants treated with water (Ctrl) or OSUB18. Data present mean ± s.e.m of three biological replicates. Data with different letters indicate a *p*-value < 0.05 on Student's t-test.

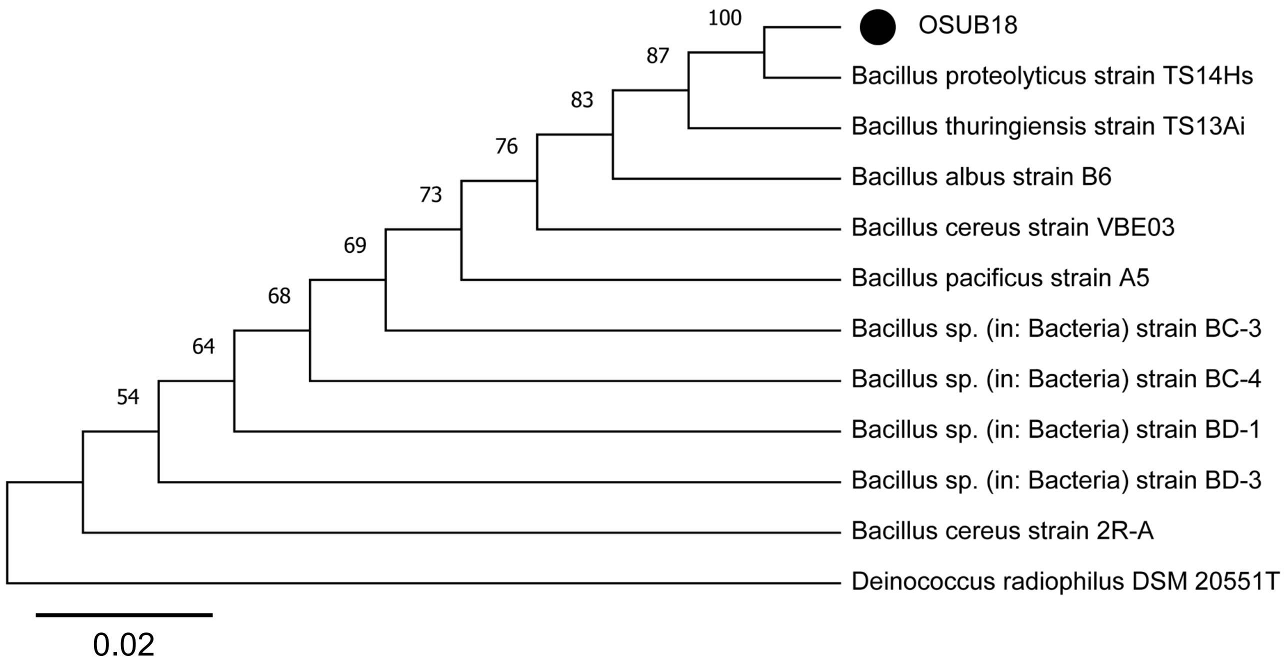

FIGURE S2

FIGURE S2 | Phylogenetic tree of OSUB18 generated by the MEGA software using the 16S rDNA sequence. The solid black dot indicates the position of OSUB18. Accession numbers of the related sequences are ON832056.1 *Bacillus thuringiensis* strain TS13Ai, ON820114.1 *Bacillus albus* strain B6, ON819722.1 *Bacillus cereus* strain VBE03, ON819625.1 *Bacillus pacificus* strain A5, ON819617.1 *Bacillus* sp. (in: Bacteria) strain BC-3, ON819616.1 *Bacillus* sp. (in: Bacteria) strain BC-4, ON819615.1 *Bacillus* sp. (in: Bacteria) strain BD-1, ON819613.1 *Bacillus* sp. (in: Bacteria) strain BD-3, ON818213.1 *Bacillus cereus* strain 2R-A, and LN681570.1 *Deinococcus radiophilus* DSM 20551T (as an out-cluster control).

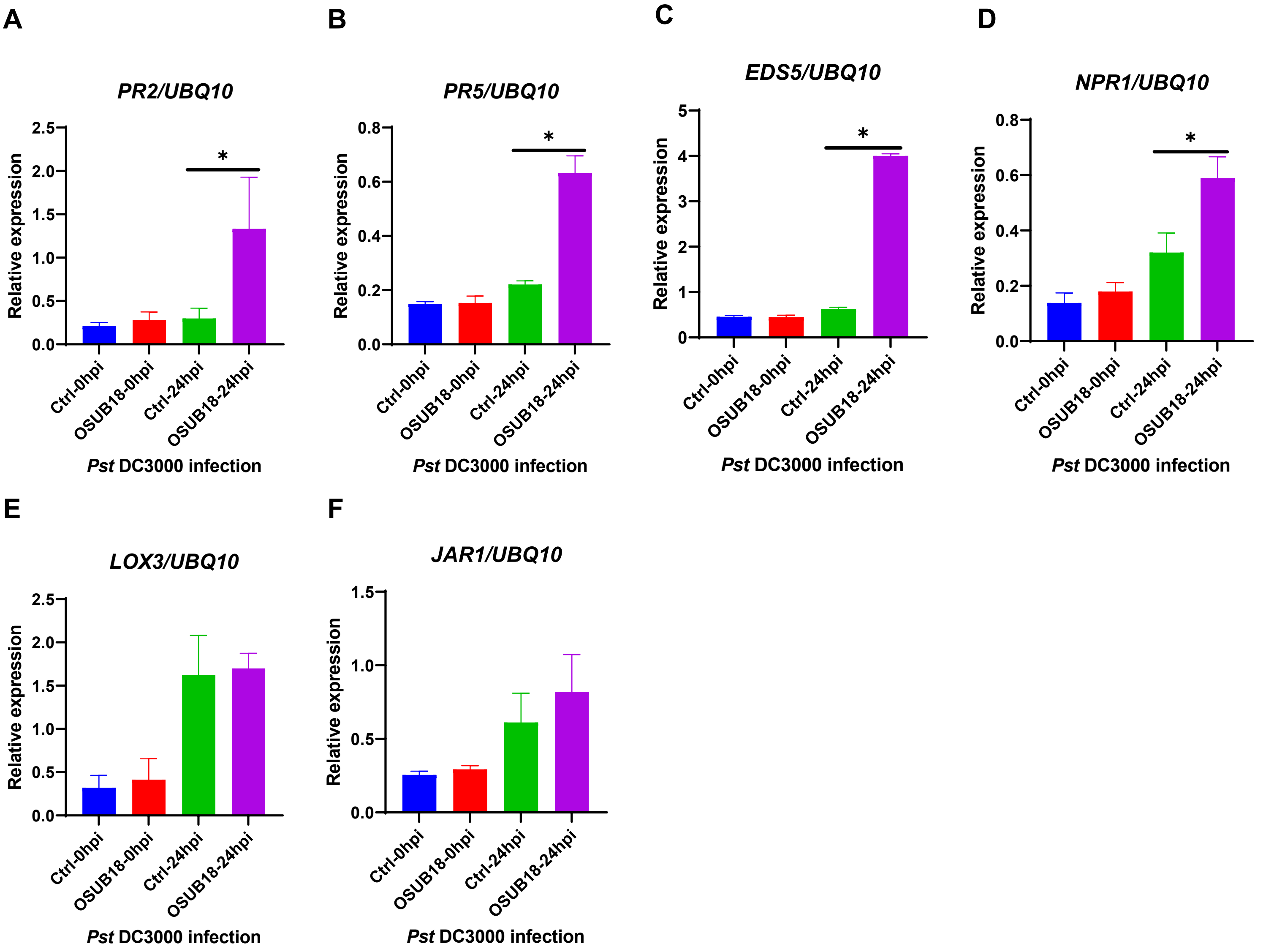

FIGURE S3

FIGURE S3 | OSUB18 root drench treatment increased the plant defense-related gene expression in *A. thaliana* after the bacterial pathogen *Pst* DC3000 infection. (A) Relative gene expression of *PR2*. **(B)** Relative gene expression of *PR5*. **(C)** Relative gene expression of *EDS5*. **(D)** Relative gene expression of *NPR1*. **(E)** Relative gene expression of *LOX3*. (F) Relative gene expression of *JAR1*. Water or OSUB18-drenched plants were infected with the *Pst* DC3000 by syringe injection. 0 or 24 hours later, the injected leaves were collected for RNA extraction and qRT-PCR assay. The *UBQ10* gene was used as an internal reference in the qRT-PCR assay. Data present mean ± SD of three biological replicates. Data with * indicate a p-value < 0.05 on Student's t-test.

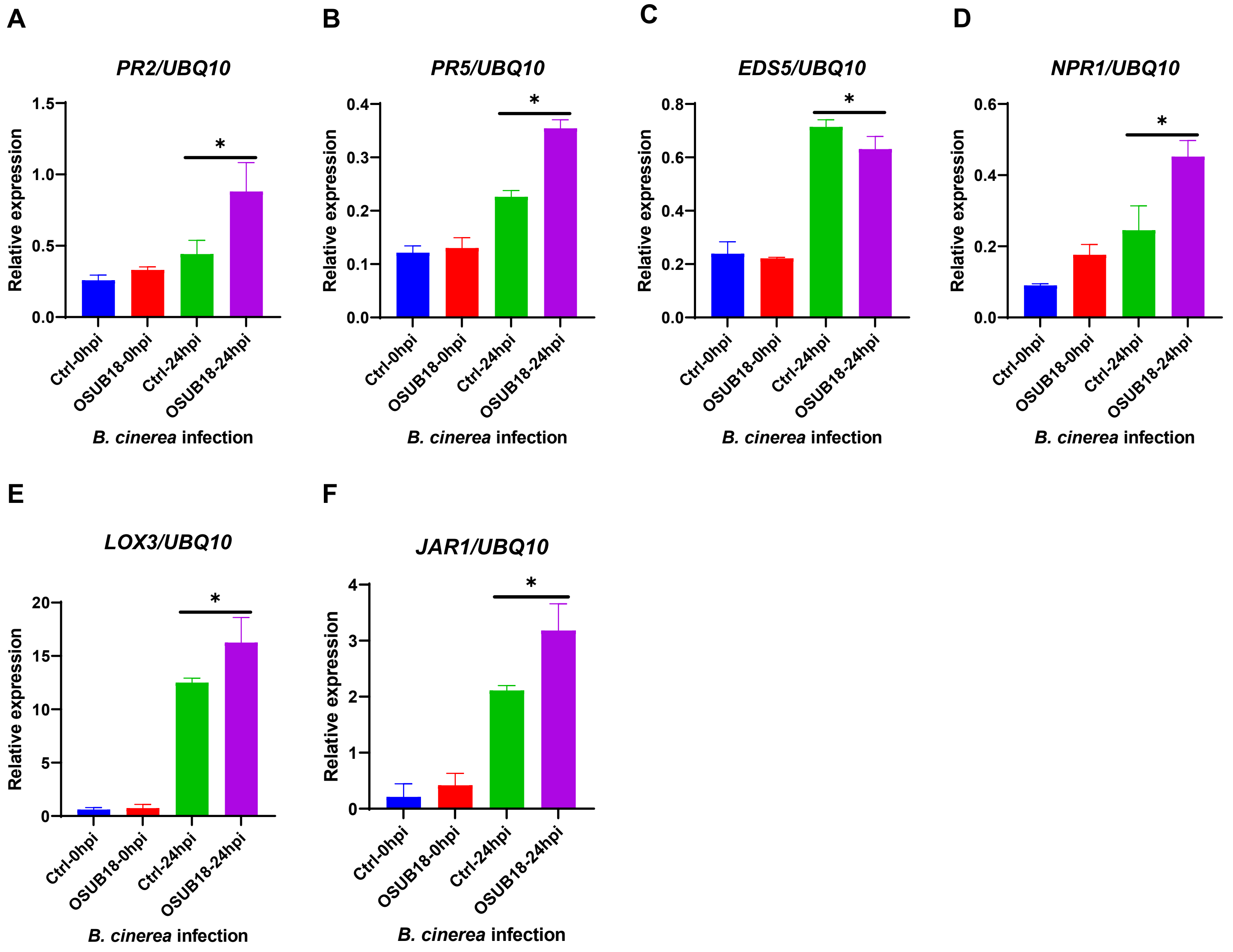

FIGURE S4

FIGURE S4 | OSUB18 root drench treatment increased the plant defense-related gene expression in *A. thaliana* after the fungal pathogen *B. cinerea* infection. (A) Relative gene expression of *PR2*. **(B)** Relative gene expression of *PR5*. **(C)** Relative gene expression of *EDS5*. **(D)** Relative gene expression of *NPR1*. **(E)** Relative gene expression of *LOX3*. **(F)** Relative gene expression of *JAR1*. Water or OSUB18-drenched plants were infected with *B. cinerea* by spore inoculation. 0 or 24 hours later, the injected leaves were collected for RNA extraction and qRT-PCR assay. The *UBQ10* gene was used as an internal reference in the qRT-PCR assay. Data present mean ± SD of three biological replicates. Data with * indicate a *p*-value < 0.05 on Student's t-test.

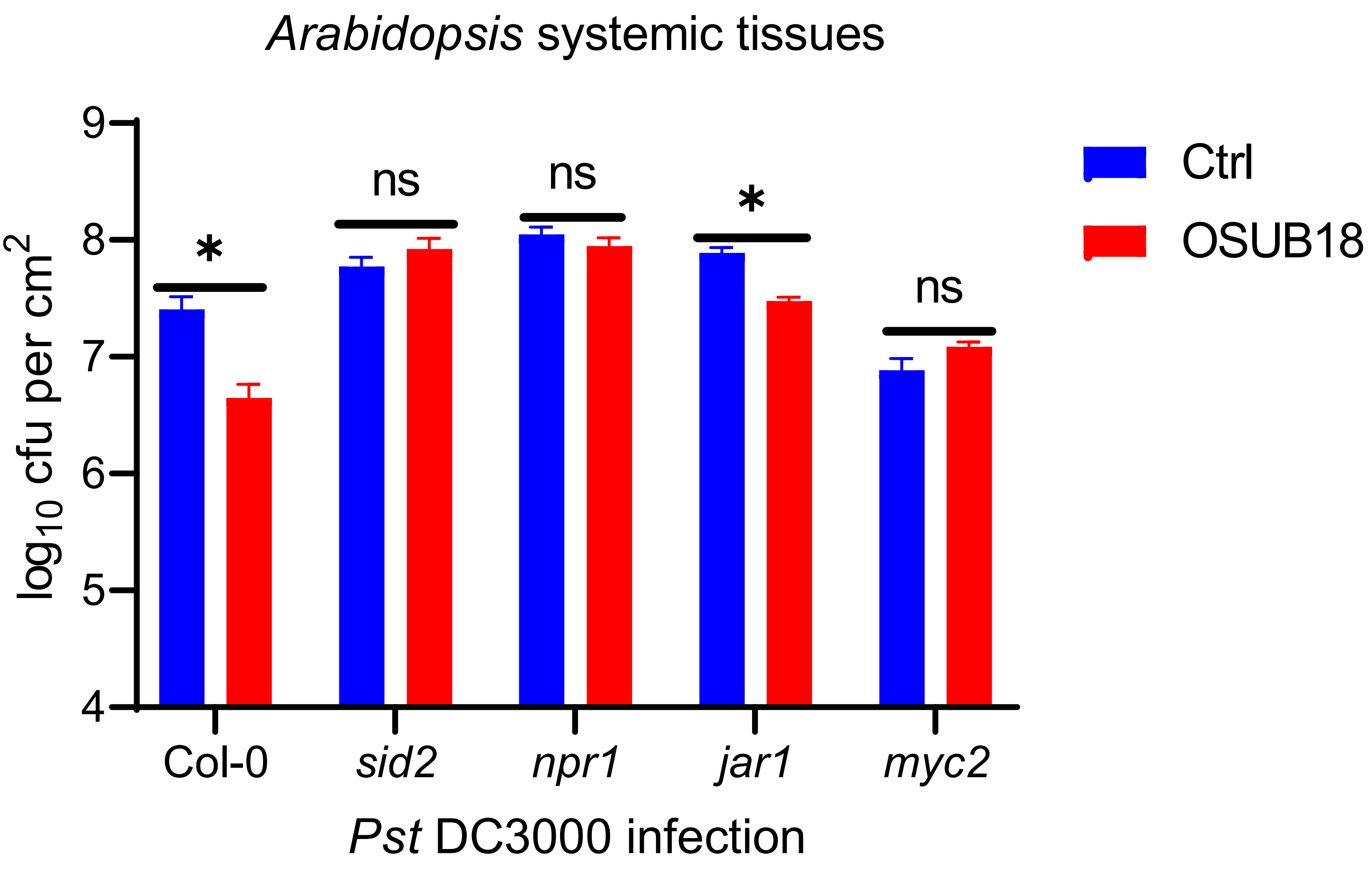

FIGURE S5

FIGURE S5 | OSUB18 root drench treatment induced systemic resistance against the bacterial pathogen *Pst* DC3000 through a *SID2*-, *NPR1*- and *MYC2*-dependent signaling pathway. Water or OSUB18-drenched Col-0 plants were infected with *Pst* DC3000 by syringe injection. The bacterial pathogen growth was examined at 3dpi. Data present mean ± s.e.m of three biological replicates. Data with * indicate a p-value < 0.05 on Student's t-test.

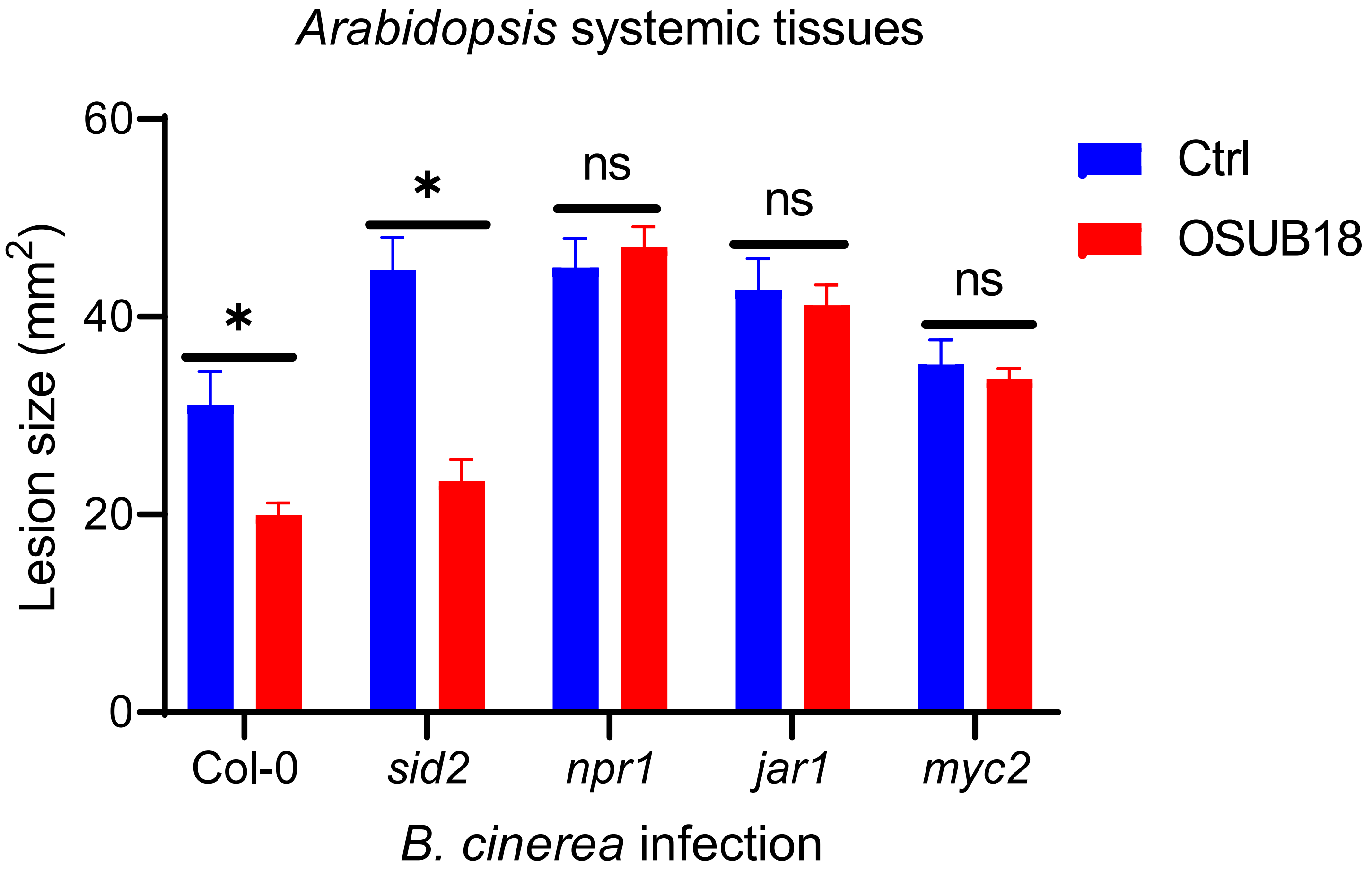

FIGURE S6

FIGURE S6 | OSUB18 root drench treatment induced systemic resistance against the fungal pathogen *B. cinerea* through an *NPR1*-, *JAR1*- and *MYC2*-dependent signaling pathway. Water or OSUB18-drenched Col-0 plants were infected with *B. cinerea* by spore inoculation. The fungal pathogen growth was examined at 3dpi. Data present mean ± s.e.m of three biological replicates. Data with * indicate a p-value < 0.05 on Student's t-test.

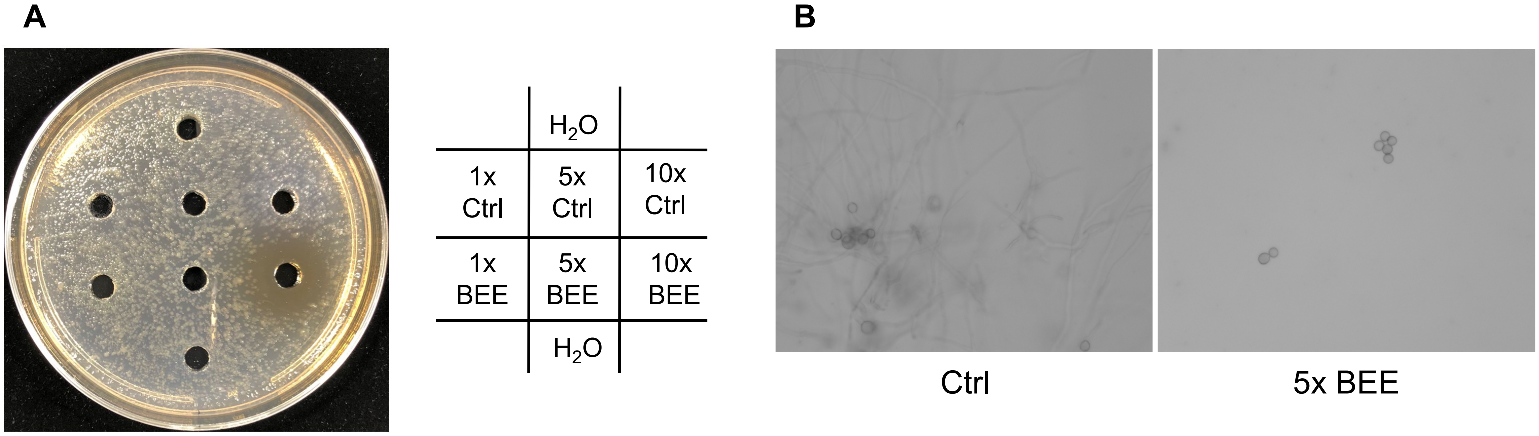

FIGURE S7

FIGURE S7 | OSUB18 metabolites inhibited bacterial and fungal pathogen growth in vitro. (A) Bacterial extracellular exudates (BEE) of OSUB18 inhibited the growth of *Pst* DC3000 on agar plates. Pst DC3000 cells (100ul, 10^8 CFU/ml) were evenly distributed on the KBA plate. The 8 oxford cups were created in the plate with sterile pipette tips. 40 µL of the indicated solution was deployed to the individual oxford cup, respectively. The inhibition zone was observed and pictured 2 days after the incubation of the KBA plates at 28°C. 10x BEE, 10x concentrated BEE of OSUB18. 5x BEE, 5xconcentrated BEE of OSUB18. 5x BEE was diluted from 10x BEE with sterile water. **(B)** OSUB18 BEE inhibited the spore germination and hypha development of B. cinerea. B. cinerea spores were collected to Ctrl (half-strength V8 juice) or 5x BEE (in half-strength V8 juice) of OSUB18 and incubated at RT for one day before the spore development was examined under a microscope.

Supplementary Table 1. Primers used in this study.

Name	Sequence (5'-3')	Purpose	Reference
799F	ACACTGACGACATGGTTCTACAAAC MGGATTAGATACCCKG	To amplify the 16S rDNA	(Chen et al., 2020)
1193R	TACGGTAGCAGAGACTTGGTCTACG TCATCCCCACCTTCC	To amplify the 16S rDNA	(Chen et al., 2020)
UBQ10F	AAAGAGATAACAGGAACGGAAACA TAGT	qRT-PCR	(Pozo et al., 2008)
UBQ10R	GGCCTTGTATAATCCCTGATGAATA AG	qRT-PCR	(Pozo et al., 2008)
PR1F	CTCGGAGCTACGCAGAACAA	qRT-PCR	(Nie et al., 2017)
PR1R	TTCTCGCTAACCCACATGTTCA	qRT-PCR	(Nie et al., 2017)
SID2F	CCAATTGACCAGCAAATCGGAGCA	qRT-PCR	(Zhao et al., 2022b)
SID2R	CGTTTCCGTTTCCGTTTCCGTTCT	qRT-PCR	(Zhao et al., 2022b)
PDF1.2F	AGTTGTGCGAGAAGCCAAGT	qRT-PCR	(Nie et al., 2017)
PDF1.2R	GTTGCATGATCCATGTTTGG	qRT-PCR	(Nie et al., 2017)
COI1F	CATGGCGGTGTATGTCTCAGA	qRT-PCR	(Zhao et al., 2022b)
COI1R	TCGAGTAAGACAAGGCGGAAGT	qRT-PCR	(Zhao et al., 2022b)
MYC2F	GATGAGGAGGTGACGGATACGGAA	qRT-PCR	(Pozo et al., 2008)
MYC2R	CGCTTTACCAGCTAATCCCGCA	qRT-PCR	(Pozo et al., 2008)
RBOHDF	AGCTTCACAATTATTGC ACGAG	qRT-PCR	(Zhao et al., 2022a)
RBOHDR	TCTCCAGTTAGGTTTA GCGAAG	qRT-PCR	(Zhao et al., 2022a)
PR2F	ATCAAGGAGCTTAGCCTCAC	qRT-PCR	(Wang et al., 2019)

PR2R	TGTAAAGAGCCACAACGTCC	qRT-PCR	(Wang et al., 2019)
PR5F	CTCTTCCTCGTGTTCATCAC	qRT-PCR	(Wang et al., 2019)
PR5R	GAAGCACCTGGAGTCAATTC	qRT-PCR	(Wang et al., 2019)
EDS5F	GGCGATGGGGATGTGGATTT	qRT-PCR	This study
EDS5R	GTACTGTTCCCGGTCCAAGA	qRT-PCR	This study
NPR1F	ACGAAGAGAACATCACCGGG	qRT-PCR	This study
NPR1R	TTCCCGAGTTCCACGGTTTT	qRT-PCR	This study
LOX3F	CGGATAGAGAAAGAGATTGAGAAA AGGAAC	qRT-PCR	(Habash et al., 2020)
LOX3R	AGGTACACCTCTACACGTAACACCA GGC	qRT-PCR	(Habash et al., 2020)
JAR1F	TGCCATTTCCTTAAGCTCTGGA	qRT-PCR	This study
JAR1R	GAAGGCAAAAGCAGTGCGAA	qRT-PCR	This study

Supplementary Table 2. Beneficial traits of OSUB18 and Pf5[1].

Assay	OSUB18	Pf5	Trait function(s)
Siderophore production	Positive	Positive	ISR[2] (Berendsen et al., 2015); PI[3] (Leong, 1986); PGP (Pahari et al., 2017)
Exopolysaccharide production	Positive	Positive	ISR (Jiang et al., 2016); PGP[4] (Naseem et al., 2018); PI (Abdalla et al., 2021)
Acetoin/diacetyl production	Positive	Negative	ISR (Peng et al., 2019); PGP (Sharifi and Ryu, 2018); PI (Kai et al., 2007)
HCN[5] production	Negative	Positive	PI (Anand et al., 2020); PGP (Rijavec and Lapanje, 2016)
Ammonia production	Positive	Positive	PI (Mota et al., 2017); PGP (Hayat et al., 2010)
IAA[6] production	Positive	Positive	PGP (Etesami et al., 2015)
Phosphate solubilization	Negative	Positive	PGP (Alori et al., 2017)
Organic acid production	Negative	Positive	PGP (Macias-Benitez et al., 2020); PI (Makras and De Vuyst, 2006)
Catalase activity	Positive	Positive	PGP (Lopes et al., 2021)

[1]Pf5: the plant commensal bacterial strain *Pseudomonas fluorescens* Pf5; [2]ISR: induced systemic resistance; [3]PI: pathogen inhibition; [4]PGP: plant growth promotion; [5]HCN: Hydrogen cyanide; [6]IAA: indole-3-acetic acid.

References

Abdalla, A. K., Ayyash, M. M., Olaimat, A. N., Osaili, T. M., Al-Nabulsi, A. A., Shah, N. P., et al. (2021). Exopolysaccharides as Antimicrobial Agents: Mechanism and Spectrum of Activity. *Frontiers in Microbiology* 12. doi: 10.3389/fmicb.2021.664395.

Alori, E. T., Glick, B. R., and Babalola, O. O. (2017). Microbial Phosphorus Solubilization and Its Potential for Use in Sustainable Agriculture. *Frontiers in Microbiology* 8. doi: 10.3389/fmicb.2017.00971.

Anand, A., Chinchilla, D., Tan, C., Mène-Saffrané, L., L'Haridon, F., and Weisskopf, L. (2020). Contribution of Hydrogen Cyanide to the Antagonistic Activity of Pseudomonas Strains Against Phytophthora infestans. *Microorganisms* 8, 1144. doi: 10.3390/microorganisms8081144.

Berendsen, R. L., van Verk, M. C., Stringlis, I. A., Zamioudis, C., Tommassen, J., Pieterse, C. M. J., et al. (2015). Unearthing the genomes of plant-beneficial Pseudomonas model strains WCS358, WCS374 and WCS417. *BMC Genomics* 16, 539. doi: 10.1186/s12864-015-1632-z.

Chen, T., Nomura, K., Wang, X., Sohrabi, R., Xu, J., Yao, L., et al. (2020). A plant genetic network for preventing dysbiosis in the phyllosphere. *Nature* 580, 653–657. doi: 10.1038/s41586-020-2185-0.

Etesami, H., Alikhani, H. A., and Hosseini, H. M. (2015). Indole-3-acetic acid (IAA) production trait, a useful screening to select endophytic and rhizosphere competent bacteria for rice growth promoting agents. *MethodsX* 2, 72–78. doi: 10.1016/j.mex.2015.02.008.

Habash, S. S., Könen, P. P., Loeschcke, A., Wüst, M., Jaeger, K.-E., Drepper, T., et al. (2020). The Plant Sesquiterpene Nootkatone Efficiently Reduces Heterodera schachtii Parasitism by Activating Plant Defense. *International Journal of Molecular Sciences* 21, 9627. doi: 10.3390/ijms21249627.

Hayat, R., Ali, S., Amara, U., Khalid, R., and Ahmed, I. (2010). Soil beneficial bacteria and their role in plant growth promotion: a review. *Annals of Microbiology* 60, 579–598.

Jiang, C.-H., Fan, Z.-H., Xie, P., and Guo, J.-H. (2016). Bacillus cereus AR156 Extracellular Polysaccharides Served as a Novel Micro-associated Molecular Pattern to Induced Systemic Immunity to Pst DC3000 in Arabidopsis. *Front. Microbiol.* 7. doi: 10.3389/fmicb.2016.00664.

Kai, M., Effmert, U., Berg, G., and Piechulla, B. (2007). Volatiles of bacterial antagonists inhibit mycelial growth of the plant pathogen Rhizoctonia solani. *Arch Microbiol* 187, 351–360. doi: 10.1007/s00203-006-0199-0.

Leong, J. (1986). Siderophores: Their Biochemistry and Possible Role in the Biocontrol of Plant Pathogens. *Annual Review of Phytopathology* 24, 187–209. doi: 10.1146/annurev.py.24.090186.001155.

Lopes, M. J. dos S., Dias-Filho, M. B., and Gurgel, E. S. C. (2021). Successful Plant Growth-Promoting Microbes: Inoculation Methods and Abiotic Factors. *Frontiers in Sustainable Food Systems* 5. doi: 10.3389/fsufs.2021.606454.

Macias-Benitez, S., Garcia-Martinez, A. M., Caballero Jimenez, P., Gonzalez, J. M., Tejada Moral, M., and Parrado Rubio, J. (2020). Rhizospheric Organic Acids as Biostimulants: Monitoring Feedbacks on Soil Microorganisms and Biochemical Properties. *Frontiers in Plant Science* 11. doi: 10.3389/fpls.2020.00633.

Makras, L., and De Vuyst, L. (2006). The in vitro inhibition of Gram-negative pathogenic bacteria by bifidobacteria is caused by the production of organic acids. *International Dairy Journal* 16, 1049–1057. doi: 10.1016/j.idairyj.2005.09.006.

Mota, M. S., Gomes, C. B., Souza Júnior, I. T., and Moura, A. B. (2017). Bacterial selection for biological control of plant disease: criterion determination and validation. *Brazilian Journal of Microbiology* 48, 62–70. doi: 10.1016/j.bjm.2016.09.003.

Naseem, H., Ahsan, M., Shahid, M. A., and Khan, N. (2018). Exopolysaccharides producing rhizobacteria and their role in plant growth and drought tolerance. *J Basic Microbiol* 58, 1009–1022. doi: 10.1002/jobm.201800309.

Nie, P., Li, X., Wang, S., Guo, J., Zhao, H., and Niu, D. (2017). Induced Systemic Resistance against Botrytis cinerea by Bacillus cereus AR156 through a JA/ET- and NPR1-Dependent Signaling Pathway and Activates PAMP-Triggered Immunity in Arabidopsis. *Front. Plant Sci.* 8. doi: 10.3389/fpls.2017.00238.

Pahari, A., Pradhan, A., Nayak, S. K., and Mishra, B. B. (2017). "Bacterial Siderophore as a Plant Growth Promoter," in *Microbial Biotechnology: Volume 1. Applications in Agriculture and Environment*, eds. J. K. Patra, C. N. Vishnuprasad, and G. Das (Singapore: Springer), 163–180. doi: 10.1007/978-981-10-6847-8_7.

Peng, G., Zhao, X., Li, Y., Wang, R., Huang, Y., and Qi, G. (2019). Engineering Bacillus velezensis with high production of acetoin primes strong induced systemic resistance in Arabidopsis thaliana. *Microbiological Research* 227, 126297. doi: 10.1016/j.micres.2019.126297.

Pozo, M. J., Ent, S. V. D., Loon, L. C. V., and Pieterse, C. M. J. (2008). Transcription factor MYC2 is involved in priming for enhanced defense during rhizobacteria-induced systemic resistance in Arabidopsis thaliana. *New Phytologist* 180, 511–523. doi: https://doi.org/10.1111/j.1469-8137.2008.02578.x.

Rijavec, T., and Lapanje, A. (2016). Hydrogen Cyanide in the Rhizosphere: Not Suppressing Plant Pathogens, but Rather Regulating Availability of Phosphate. *Front. Microbiol.* 0. doi: 10.3389/fmicb.2016.01785.

Sharifi, R., and Ryu, C.-M. (2018). Revisiting bacterial volatile-mediated plant growth promotion: lessons from the past and objectives for the future. *Ann Bot* 122, 349–358. doi: 10.1093/aob/mcy108.

Wang, C., Huang, X., Li, Q., Zhang, Y., Li, J.-L., and Mou, Z. (2019). Extracellular pyridine nucleotides trigger plant systemic immunity through a lectin receptor kinase/BAK1 complex. *Nature Communications* 10, 1–16. doi: 10.1038/s41467-019-12781-7.

Zhao, Z., Fan, J., Gao, Y. G., Wang, Z., Yang, P., Liang, Y., et al. (2022a). Arabidopsis Plasma Membrane ATPase AHA5 Is Negatively Involved in PAMP-Triggered Immunity. *International Journal of Molecular Sciences* 23, 3857. doi: 10.3390/ijms23073857.

Zhao, Z., Fan, J., Yang, P., Wang, Z., Stephen, O., Mackey, D., et al. (2022b). Involvement of Arabidopsis Acyl Carrier Protein 1 in PAMP-triggered immunity. *MPMI*. doi: 10.1094/MPMI-02-22-0049-R.
